# Early Initiation of Dapagliflozin in Acute Heart Failure: A Systematic Review and Meta-Analysis of Randomized Controlled Trials

**DOI:** 10.3390/biomedicines14102319

**Published:** 2026-10-13

**Authors:** Abdullah Almaqhawi, Ahmed K. Alsaif, Hamza Ashraf, Miad Ali Alhamrani, Nada Khaled Ameen, Abdulaziz Turki Alqabli, Lama Khalid Almatrfi, Saud Hassan S. Hussain, Lina Fahad Bahitham, Juwadaa S. Alnajjar, Ali Ahmed K. Albahrani

**Affiliations:** 1 Department of Family Medicine and Community, College of Medicine, King Faisal University, Al-Ahsa 36362, Saudi Arabia; 2 College of Medicine, Al-Rayan Colleges, Al-Madinah 42541, Saudi Arabia; a.k.alsaif.md@gmail.com; 3 Department of Medicine, Allama Iqbal Medical College, Lahore 54550, Pakistan; a93.hamza@gmail.com; 4 College of Medicine, University of Jeddah, Jeddah 23218, Saudi Arabia; miad.ali.alhamrani@gmail.com (M.A.A.); nadaameen477@gmail.com (N.K.A.); 5 Faculty of Medicine, University of Tabuk, Tabuk 71491, Saudi Arabia; abdulaziz.alqabli@gmail.com; 6 College of Medicine, Umm Al-Qura University, Makkah 21955, Saudi Arabia; lamaabntkhalid@gmail.com (L.K.A.); lina.fahad.ba@gmail.com (L.F.B.); 7 College of Medicine, King Khalid University, Abha 62521, Saudi Arabia; saudhussain30@gmail.com; 8 Academy of Family Medicine, Ministry of Health, Al-Ahsa 36361, Saudi Arabia; jawdaa22@gmail.com; 9 Al-Salhyiah Primary Healthcare Center, Al-Ahsa Health Cluster, Ministry of Health, Al-Ahsa 36441, Saudi Arabia; wmu.drali@gmail.com

**Keywords:** dapagliflozin, SGLT2 inhibitors, acute heart failure, meta-analysis, systematic review

## Abstract

**Highlights:**

**What are the main findings?**

- Early dapagliflozin initiation significantly reduced all-cause mortality but not heart failure hospitalization in the all-population analysis.
- Initiation of dapagliflozin was associated with a significant reduction in urinary tract infections (UTIs), without an increase in any adverse events.

**What are the implications of the main findings?**

- The available randomized evidence supports short-term safety with a clear all-population mortality.
- Adequately powered AHF-specific trials with prespecified timing strata are needed before definitive clinical recommendations can be made.

**Abstract:**

**Background/Objectives:** Dapagliflozin is a foundational chronic heart failure therapy, but whether it retains benefit when initiated early during hospitalization for acute heart failure (AHF) remains uncertain. **Methods:** We searched MEDLINE, Embase, Scopus, and CENTRAL from inception to 26 January 2026 for randomized trials comparing early initiation of dapagliflozin 10 mg once daily with standard therapy in adults hospitalized for AHF (PROSPERO CRD420261364466). Two reviewers independently performed screening, extraction, and risk-of-bias assessment. Outcomes were pooled under random-effects models with Knapp–Hartung adjustment. **Results:** Ten trials enrolling 3507 participants (1769 in the dapagliflozin group and 1738 in the control group) were eligible. Dapagliflozin significantly reduced all-cause mortality (RR 0.67, 95% CI 0.52–0.86) and urinary tract infections (RR 0.52, 95% CI 0.35–0.76), while no significant differences were observed in cardiovascular death, MACEs, heart failure hospitalization, all-cause readmission, length of hospital stay, or body weight. Dapagliflozin was not associated with a significant increase in acute kidney injury, hypotension, or hypoglycemia, supporting a favorable safety profile. **Conclusions:** In adults hospitalized with acute heart failure (AHF), dapagliflozin significantly reduced all-cause mortality but not heart failure hospitalization during follow-up. Larger, adequately powered trials in patients with AHF, with prespecified analyses according to the timing of dapagliflozin initiation, are warranted.

## 1. Introduction

Acute heart failure (AHF) is a life-threatening clinical syndrome characterized by new-onset or worsening signs and symptoms of heart failure that are severe enough to prompt urgent evaluation and commonly lead to unplanned hospital admission. It remains a leading driver of hospitalization among older adults and carries a heavy burden of morbidity, mortality, and healthcare utilization, with vulnerability to adverse events persisting through admission and well beyond discharge [1].

Current in-hospital AHF management prioritizes the rapid identification and treatment of congestion and precipitating factors, most often using intravenous loop diuretics, while maintaining balance in perfusion, renal function, and hemodynamic stability. However, AHF has long been therapeutically frustrating: multiple randomized trials of in-hospital pharmacologic interventions have not consistently translated into improved post-discharge outcomes. This underscores the need for therapies that are both feasible to start during admission and able to modify early and longer-term risk [1,2]. Hospitalization provides a time-limited opportunity to initiate or intensify evidence-based, disease-modifying heart failure therapies and to structure early post-discharge follow-up, which contemporary guidelines emphasize as a strategy to reduce re-hospitalization and death [3,4].

Sodium–glucose cotransporter-2 (SGLT2) inhibitors, especially dapagliflozin, have emerged as foundational disease-modifying therapies across the heart failure spectrum, expanding well beyond their original glucose-lowering indications. In the DAPA-HF trial, dapagliflozin reduced the risk of the primary composite outcome of worsening heart failure or cardiovascular death in patients with symptomatic heart failure with reduced ejection fraction (HFrEF), with consistent benefits irrespective of the diabetes status [5]. In the DELIVER trial, dapagliflozin reduced the combined risk of worsening heart failure or cardiovascular death in patients with heart failure and mildly reduced or preserved ejection fraction (HFmrEF/HFpEF) [6]. A prespecified patient-level pooled meta-analysis of DAPA-HF and DELIVER further demonstrated reductions in cardiovascular death, all-cause mortality, and total heart failure hospitalizations across a broad range of ejection fractions, supporting a clinically meaningful and mechanistically plausible role for dapagliflozin in heart failure risk reduction as SGLT2 inhibitors promote osmotic diuresis and natriuresis, reduce cardiac preload and afterload, along with improving cardiorenal function independent of glucose lowering [7,8]. These data have informed guideline recommendations specifically supporting dapagliflozin and other SGLT2 inhibitors as a core component of contemporary heart failure pharmacotherapy, including HFmrEF and HFpEF [3,4].

Whether the clinical benefits of dapagliflozin can be preserved or even amplified when initiated early during hospitalization for AHF is a clinically important question. Early initiation could theoretically address two pressing problems in AHF care: (a) congestion and diuretic responsiveness during admission and (b) the heightened early post-discharge risk period, when therapeutic inertia, suboptimal initiation of guideline-directed medical therapy, and limited follow-up may contribute to recurrent adverse events [3,4]. Simultaneously, clinicians have legitimate safety concerns regarding the initiation of SGLT2 inhibition during acute illness, including risks associated with fluctuating volume status, blood pressure, renal function, and the rare but serious complication of ketoacidosis in susceptible patients [4]. These concerns underscore the need for trial-level evidence, particularly randomized comparisons with meticulous adverse event ascertainment, to inform decision-making.

Evidence supporting early in-hospital SGLT2 inhibitor initiation has expanded in recent years, providing proof-of-concept for the feasibility in stabilized AHF. In the EMPULSE study, initiation of empagliflozin in clinically stabilized patients hospitalized for acute de novo or decompensated chronic heart failure improved a hierarchical 90-day clinical benefit outcome and was well tolerated, suggesting that SGLT2 inhibition can be safely started during AHF hospitalization under appropriate stability criteria [2]. In the SOLOIST-WHF trial, sotagliflozin initiated before discharge or shortly after discharge in patients with diabetes and recent worsening heart failure reduced total cardiovascular deaths and heart failure events compared with placebo, reinforcing the concept that early post-acute initiation may yield clinically relevant benefits [9]. However, when considering dapagliflozin specifically in the AHF setting, available evidence is comparatively limited and heterogeneous, often focusing on short-term decongestion and safety rather than mortality.

Several randomized trials have directly informed the early use of dapagliflozin in AHF-relevant inpatient scenarios. DICTATE-AHF evaluated dapagliflozin initiated during hospitalization among patients with acute heart failure receiving intravenous loop diuretics and provided important randomized evidence regarding its physiological effects and safety in this setting [10]. DAPA-RESIST compared dapagliflozin with metolazone as an add-on strategy in hospitalized patients with heart failure and loop diuretic resistance; dapagliflozin was not more effective at relieving congestion than metolazone but was associated with less biochemical disturbance, a potentially meaningful safety/tolerability advantage in a high-risk inpatient population [11]. More recently, DAPA ACT HF–TIMI 68 tested in-hospital initiation of dapagliflozin in stabilized acute heart failure hospitalization and, based on publicly reported primary results, did not demonstrate a statistically significant reduction in cardiovascular death or worsening heart failure through short-term follow-up, although the evolving totality of randomized evidence continues to inform the magnitude and certainty of benefit for early in-hospital SGLT2 initiation [12,13].

Given the rapid emergence of randomized trials with differing eligibility criteria (e.g., stabilization requirements and diuretic resistance), timing of initiation, follow-up duration, and outcome definitions, the net clinical effect and safety profile of early dapagliflozin initiation in AHF remain uncertain when considering single trials in isolation. Therefore, a focused synthesis of randomized evidence is needed to quantify the potential benefits and harms, improve the precision of mortality and safety outcomes, and clarify the consistency across trial designs and patient subgroups. This systematic review and meta-analysis aimed to evaluate whether early initiation of dapagliflozin compared with control or conventional therapy improves clinically relevant outcomes, including all-cause mortality, heart failure rehospitalization, composite heart failure events, and cardiovascular and non-cardiovascular adverse events in adult patients hospitalized with acute heart failure.

## 2. Methods

This systematic review and meta-analysis were performed in line with the PRISMA guidelines and the Cochrane Handbook for Systematic Reviews of Interventions [14,15]. PRISMA 2020 checklist is included in Supplementary Table S1. The protocol was registered with PROSPERO (CRD420261364466). As the study relied on previously published data, ethical approval was not required.

### 2.1. Search Strategy and Data Sources

A comprehensive literature search was carried out across multiple electronic databases to identify studies assessing the efficacy and safety of dapagliflozin in patients with acute heart failure. The databases included MEDLINE (via PubMed), Embase (via Ovid), Scopus, and the Cochrane Central Register of Controlled Trials. A combination of MeSH terms and free-text keywords related to “SGLT2 inhibitors,” “dapagliflozin,” “acute heart failure,” and “randomized controlled trials” was applied. Reference lists of relevant articles and review papers were also screened to capture any additional studies. The search covered all records published from inception up to 26 January 2026. Detailed search strategies for each database are presented in Supplementary Table S2.

### 2.2. Selection Process

Two reviewers (Miad Ali Alhamrani and Lina Fahad Bahitham) independently screened the titles and abstracts of all retrieved records. Studies that met the eligibility criteria, or that could not be confidently judged from the title and abstract alone, were advanced to full-text review. Disagreements were resolved by discussion, with a third senior reviewer (Ahmed Alsaif) consulted where consensus could not be reached.

### 2.3. Inclusion and Exclusion Criteria

Studies were eligible for inclusion if they met the following criteria: (1) randomized controlled trials; (2) comparison of dapagliflozin (10 mg once daily) with standard or conventional therapy; (3) evaluation of dapagliflozin initiated on hospitalization or within days after admission for acute heart failure (4) reporting of at least one predefined outcome of interest; and (5) publication in any language. No minimum sample size was required for inclusion. We excluded non-randomized studies, retrospective cohort studies, case reports, editorials, non-human studies, and meeting abstracts.

### 2.4. Data Collection

A standardized data extraction form was developed to ensure consistent capture of key variables across all included studies. Two authors independently extracted the data using the standardized form, with discrepancies resolved through discussion. The form included essential study characteristics such as study design, year, country, trial identifier, sample size, and duration of follow-up. Participant-level data included mean age, sex distribution, baseline comorbidities such as type 2 diabetes, hypertension, and ischemic heart disease, along with key clinical measures including left ventricular ejection fraction and estimated glomerular filtration rate. Information on the timing of dapagliflozin initiation was also recorded. When data were incomplete or unclear, study authors were contacted for clarification. If no response was received, data were extracted from graphs using PlotDigitizer [16].

### 2.5. Outcome and Subgroups

All-cause mortality was considered the primary outcome of interest. Secondary outcomes included major adverse cardiovascular events (MACEs; a composite of cardiovascular death, myocardial infarction, and stroke), individual cardiovascular outcomes (cardiovascular death and stroke), length of hospital stay, readmission, and heart failure-related hospitalization. Functional outcomes included changes in body weight in kg from admission to final follow-up, serum creatinine, estimated glomerular filtration rate (eGFR). Safety outcomes comprised acute kidney injury or worsening renal function, hypotension, urinary tract infections, and hypoglycemia. A prespecified subgroup analysis examined the effect of the timing of dapagliflozin initiation, where data permitted, comparing initiation within 12 h of hospital presentation with initiation after 12 h but within 24 h.

### 2.6. Quality Assessment

Two reviewers independently assessed the methodological quality of the included randomized trials using the Cochrane Risk of Bias tool version 2 (RoB 2), with any disagreements resolved through discussion with a third reviewer. The RoB 2 framework evaluates five domains to determine the overall risk of bias, including bias arising from the randomization process, deviations from intended interventions, missing outcome data, outcome measurement, and selective reporting of results.

### 2.7. Statistical Analysis

All analyses were performed using RC MetaStudio v0.2.3, an open-source desktop application for conducting meta-analyses through a graphical interface [17]. For continuous outcomes, mean differences (MDs) with 95% confidence intervals (CIs) were calculated. When available, data reported as means and standard deviations (SDs) were directly extracted. For studies reporting medians and interquartile ranges (IQRs), medians were converted to means and IQRs were transformed into SDs using the methods described by Luo et al. [18] and Wan et al. [19], respectively. For dichotomous outcomes, risk ratios (RRs) with 95% CIs were computed and pooled using the Mantel–Haenszel method [20] under a random-effects model. Continuous outcomes were pooled using the inverse variance method. The Knapp–Hartung adjustment was applied to the confidence intervals to account for uncertainty in the between-study variance estimate [21]. Between-study variance was estimated using the Paule–Mandel estimator [22] for dichotomous outcomes and the restricted maximum likelihood (REML) method [23] for continuous outcomes. Heterogeneity was assessed using the Higgins I^2^ statistic and interpreted according to the Cochrane Handbook guidelines, alongside the Chi^2^ test, as follows: 0–40% indicating low heterogeneity, 30–60% moderate heterogeneity, 50–75% substantial heterogeneity, and 75–100% considerable heterogeneity [24]. Publication bias was not assessed because none of the outcomes included 10 or more studies [25]. When more than two studies were available, sensitivity analyses were conducted by sequentially omitting one study at a time. A *p*-value < 0.05 was considered statistically significant for all analyses.

### 2.8. Certainty of Evidence

Grading of Recommendations Development, Assessment, and Evaluation (GRADE) approach was used to grade the certainty of evidence. The summary of the effects was generated via the GRADEpro Guideline Development Tool (Supplementary Table S3).

## 3. Results

### 3.1. Search Results

**Figure 1:**
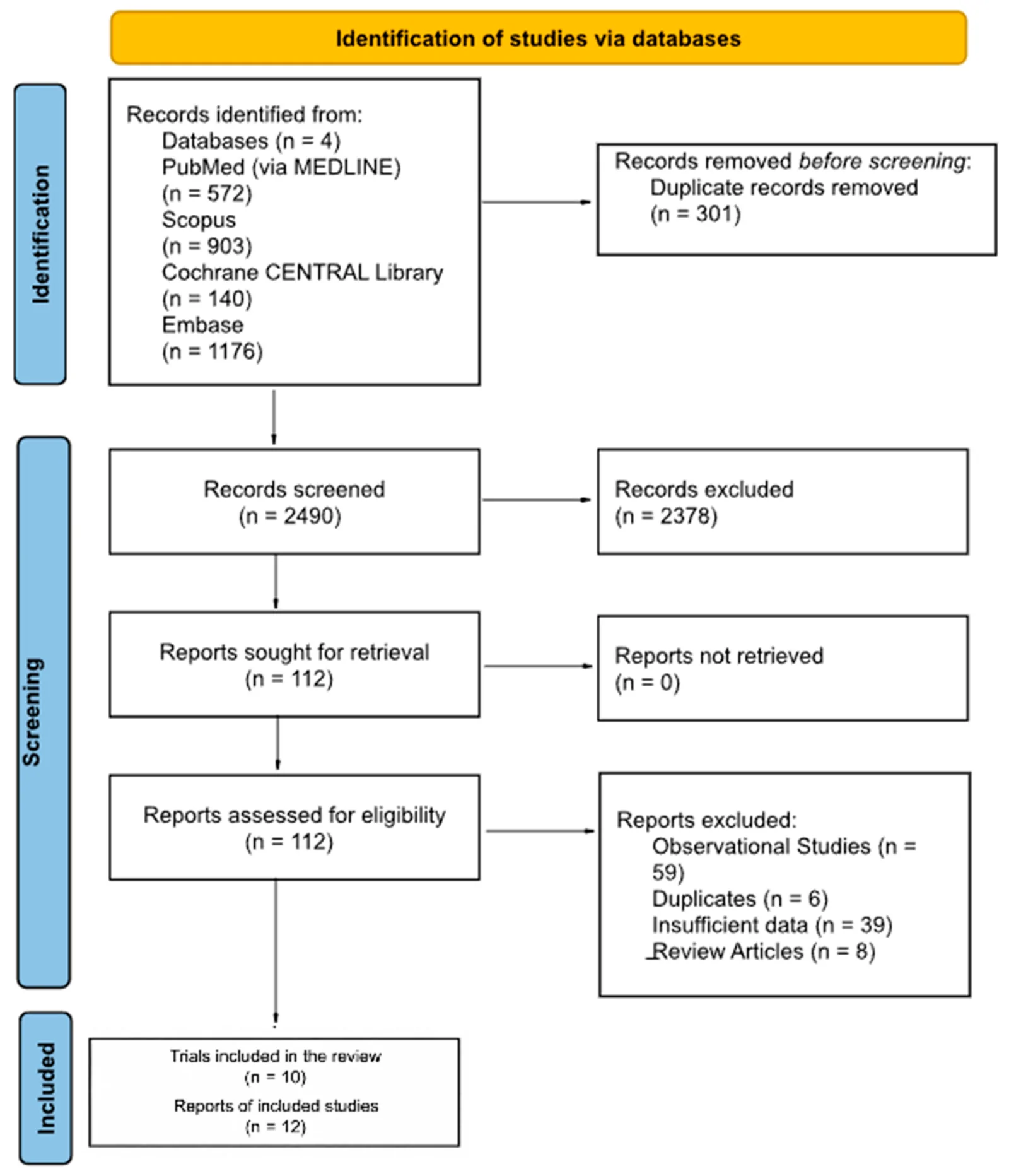
PRISMA flow diagram of study selection.

The systematic search identified 2791 articles (Figure 1). A total of 301 duplicates were removed, leaving 2490 articles for screening. Titles and abstracts were reviewed, resulting in the exclusion of 2378 records. The full texts of the remaining 112 reports were reviewed and assessed for eligibility. Ultimately, 10 RCTs reported in 12 publications, comprising 3507 participants, were eligible, while studies involving acute myocardial infarction or late initiation of dapagliflozin during or after hospitalization were excluded [10,11,12,26,27,28,29,30,31,32,33,34,35,36,37]. A total of 1769 participants were included in the dapagliflozin group, while 1738 participants were included in the control group. The trials were conducted across several countries, with two conducted in Egypt, two in the United States, and one in Russia. Single trials were conducted in Romania, Japan, Thailand, China, and the United Kingdom. Participants’ mean age ranged from 60 to 79 years. All studies administered 10 mg of dapagliflozin once daily to the patients. Baseline characteristics of studies and participants are provided in Table 1 and Table 2.

### 3.2. Quality Assessment Results

Risk of bias was evaluated in five domains: randomization process (D1), deviations from intended interventions (D2), missing outcome data (D3), outcome measurement (D4), and selective reporting of results (D5). Among the 15 included trials, 9 were judged to have “some concerns” of bias, most notably in Domain 2 and Domain 4, and 6 studies were assessed as having a “low risk” of bias. A visual summary is presented in Supplementary Figures S1 and S2.

### 3.3. Efficacy Outcomes

#### 3.3.1. Major Adverse Cardiovascular Events (MACEs)

**Figure 2:**
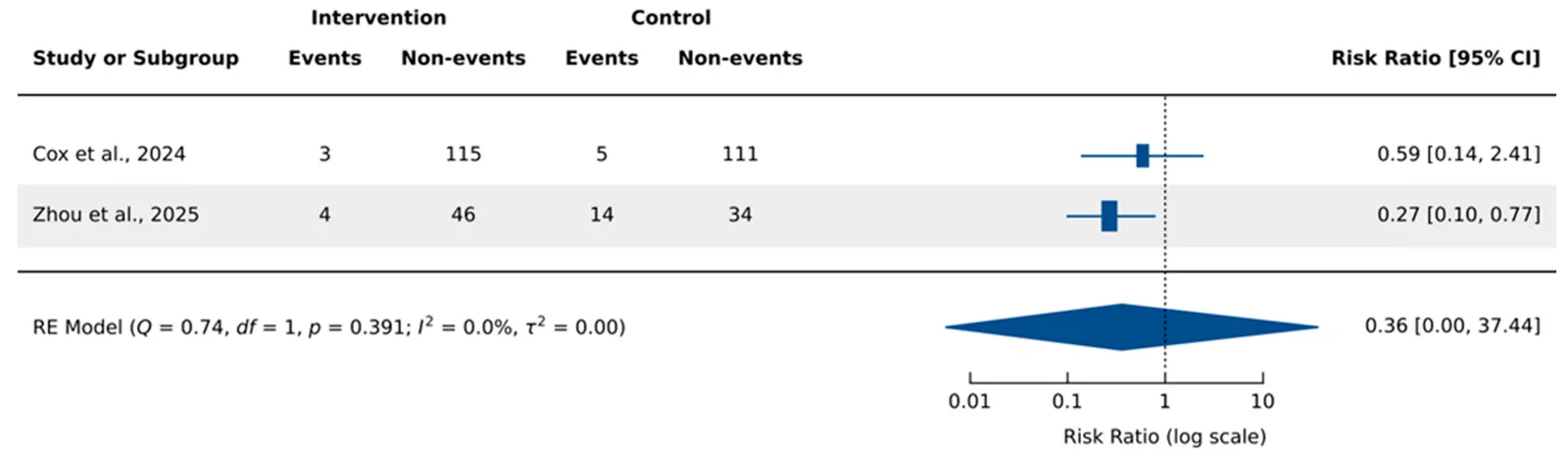
Forest plot for major adverse cardiovascular events (MACEs) [10,37].

In the pooled analysis of 2 studies [10,37], dapagliflozin did not confer a statistically significant reduction in MACEs compared with control (RR = 0.36 [0.00, 37.44]; *p* = 0.41; I^2^ = 0%) (Figure 2).

#### 3.3.2. Cardiovascular Death

In the pooled analysis of 2 studies [10,12], dapagliflozin was not associated with a significantly lower risk of cardiovascular death compared with control (RR = 0.73 [0.02, 29.49]; *p* = 0.67; I^2^ = 4.8%) (Supplementary Figure S3).

#### 3.3.3. All-Cause Mortality

**Figure 3:**
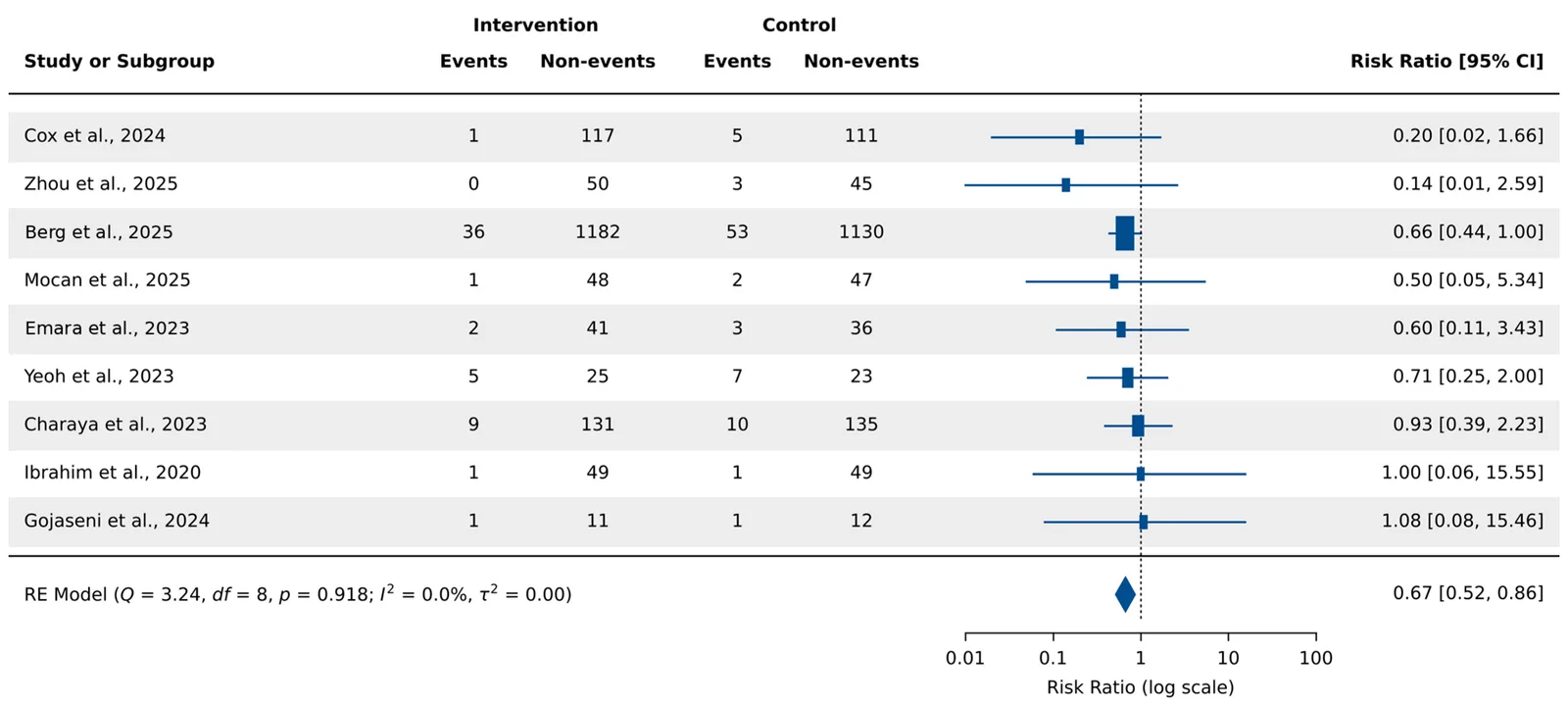
Forest plot for all-cause mortality [10,11,12,26,28,31,34,36,37].

Across 9 studies [10,11,12,26,28,31,34,36,37], dapagliflozin significantly reduced all-cause mortality relative to control (RR = 0.67 [0.52, 0.86]; *p* = 0.006; I^2^ = 0.0%) (Figure 3). Sensitivity analysis showed that this finding remained consistent after exclusion of Yeoh et al. (Supplementary Figure S4).

#### 3.3.4. Stroke

Across 2 studies [10,37], dapagliflozin was not associated with a statistically significant reduction in stroke compared with control (RR = 0.61 [0.00, 703.30]; *p* = 0.42; I^2^ = 0.0%) (Supplementary Figure S5).

#### 3.3.5. Heart Failure Hospitalization

In the pooled analysis of 4 studies [10,11,12,37], dapagliflozin was associated with a numerically lower risk of heart failure hospitalization; however, the difference did not reach statistical significance (RR = 0.87 [0.59, 1.29]; *p* = 0.3; I^2^ = 0.0%) (Supplementary Figure S6). Leave-one-out sensitivity analysis confirmed that this non-significant result was consistent across all study exclusions (Supplementary Figure S7).

#### 3.3.6. Readmission (All-Cause)

Across 7 studies [10,11,12,28,31,34,37], dapagliflozin did not significantly reduce all-cause readmission compared with control (RR = 0.78 [0.49, 1.22]; *p* = 0.217; I^2^ = 59.0%) (Supplementary Figure S8). Sensitivity analysis demonstrated that omission of Zhou et al., 2025 markedly reduced heterogeneity (I^2^ = 0%), while the results remained non-significant (RR = 0.88 [0.73, 1.06]). (Supplementary Figure S9).

#### 3.3.7. Length of Hospital Stay

In the pooled analysis of 8 studies [10,11,26,28,31,34,35,36], dapagliflozin did not significantly shorten the length of hospital stay compared with control (MD = −0.45 days [−0.96, 0.06]; *p* = 0.07; I^2^ = 14.4%) (Supplementary Figure S10). Leave-one-out sensitivity analysis showed that this finding remained non-significant across all study exclusions (Supplementary Figure S11).

### 3.4. Functional Outcomes

#### 3.4.1. Weight Change

Across 6 studies [10,11,26,31,34,35], dapagliflozin was not associated with a significantly greater reduction in body weight compared with control (MD = −0.59 kg [−1.43, −0.26]; *p* = 0.134; I^2^ = 97.2%) (Supplementary Figure S12). Although between-study heterogeneity was substantial, leave-one-out sensitivity analysis did not alter the statistical significance of the pooled estimate (Supplementary Figure S13).

#### 3.4.2. eGFR Change

The pooled analysis of 4 studies [11,32,34,35] showed no statistically significant difference in eGFR change between dapagliflozin and control (MD = −1.93 mL/min [−11.19, 7.34]; *p* = 0.32; I^2^ = 97.6%) (Supplementary Figure S14). Removal of K. Charaya et al. [31,32,33] 2023 yielded a significant result with low heterogeneity (MD = +2.55 mL/min [0.72, 4.38]; *p* = 0.027; I^2^ = 0.0%) (Supplementary Figure S15).

#### 3.4.3. Serum Creatinine

Across 2 studies [26,34], dapagliflozin did not significantly affect serum creatinine compared with control (MD = −1.71 µmol/L [−24.96, 21.54]; *p* = 0.3499; I^2^ = 97.8%) (Supplementary Figure S16).

### 3.5. Safety Outcomes

#### 3.5.1. Acute Kidney Injury (AKI)

In the pooled analysis of 8 studies [10,12,26,28,33,34,35,36], dapagliflozin was not associated with a statistically significant increase in AKI risk compared with control (RR = 1.19 [0.87, 1.64]; *p* = 0.236; I^2^ = 4.4%) (Supplementary Figure S17). Leave-one-out sensitivity analysis confirmed the robustness of this finding (Supplementary Figure S18).

#### 3.5.2. Urinary Tract Infection (UTI)

Across 5 studies [10,11,35,36,37], dapagliflozin significantly reduced the risk of urinary tract infection compared with control (RR = 0.52 [0.35, 0.76]; *p* = 0.009; I^2^ = 0.0%) (Supplementary Figure S19). This finding remained unchanged in leave-one-out sensitivity analysis (Supplementary Figure S20).

#### 3.5.3. Hypoglycemia

Across 4 studies [10,12,28,35], no statistically significant difference in hypoglycemia was observed between dapagliflozin and control (RR = 0.73 [0.41, 1.29]; *p* = 0.179; I^2^ = 0.0%) (Supplementary Figure S21). Leave-one-out sensitivity analysis showed that this finding remained stable (Supplementary Figure S22).

#### 3.5.4. Hypotension

In the pooled analysis of 7 studies [10,11,12,28,33,36,37], dapagliflozin was not associated with a statistically significant increase in hypotension risk (RR = 1.15 [0.58, 2.24]; *p* = 0.639; I^2^ = 13.9%) (Supplementary Figure S23). Leave-one-out sensitivity analysis showed that this finding remained stable (Supplementary Figure S24).

### 3.6. Subgroup Analyses Based on Timing of Dapagliflozin Introduction

#### Twelve-Hour Threshold

Subgroup analysis according to a 12 h initiation threshold was performed for length of hospital stay and weight change (Supplementary Figures S25 and S26). For length of hospital stay, no significant subgroup (*p* = 0.600) difference was observed between initiation at ≤12 h (MD = −0.20 days [−1.82, 1.42]) and initiation after 12 h but within 24 h (MD = −0.49 days [−1.43, 0.45]; *p* = 0.06). A non-significant subgroup difference was observed for weight change (*p* = 0.139), with greater weight reduction in the ≤12 h subgroup (MD = −1.38 kg [−3.60, −0.84]) than in the >12 h subgroup (MD = −0.41 kg [−1.38, −0.56]).

## 4. Discussion

This review extends the existing literature by providing the first meta-analysis focused specifically on dapagliflozin, rather than SGLT2 inhibitors as a class, in acute heart failure. It also prespecified treatment initiation timing as a potential effect modifier, incorporated smaller trials evaluating early dapagliflozin initiation that were underrepresented in previous pooled analyses and evaluated the robustness of findings through leave-one-out sensitivity analyses for all outcomes.

### 4.1. Main Findings

In this systematic review and meta-analysis of 10 randomized controlled trials enrolling 3507 participants, dapagliflozin 10 mg once daily, initiated during hospitalization significantly reduced all-cause mortality, the prespecified primary outcome.

Across the all-population analyses, no significant differences were observed for cardiovascular death, MACEs, all-cause readmission, or length of hospital stay. Heart failure hospitalization showed a directionally favourable but non-significant point estimate (RR 0.87, 95% CI 0.59–1.29) consistent with both a null effect and a clinically meaningful reduction of approximately one third, and the included trials were collectively underpowered for this endpoint. Dapagliflozin did not produce a significant reduction in body weight that, in the acute setting, is best interpreted as a surrogate marker of enhanced decongestion rather than adipose loss. No statistically significant excess was observed for acute kidney injury, hypotension, or hypoglycemia, although several of these analyses are precision-limited. The diabetic ketoacidosis analysis, based on only two trials, is uninformative (see Section 4.4). Dapagliflozin significantly reduced the risk of urinary tract infection compared with control.

This synthesis is conceptually distinct from the meta-analysis reported alongside the DAPA ACT HF–TIMI 68 primary results [12], which pooled SGLT2 inhibitors as a class in patients hospitalized for heart failure. The present analysis (i) is restricted to dapagliflozin specifically, allowing molecule-specific inference; (ii) prespecifies timing of initiation as an effect modifier; and (iii) reports leave-one-out sensitivity analyses for every outcome. The certainty of evidence was formally graded using the GRADE framework.

### 4.2. Comparison with Previous Literature

In DAPA-HF, dapagliflozin reduced the composite of worsening heart failure or cardiovascular death in patients with heart failure and reduced ejection fraction [5], and in DELIVER, analogous benefits extended into the mildly reduced and preserved ejection fraction range [6], with a patient-level pooled analysis demonstrating reductions in cardiovascular and all-cause mortality across the ejection fraction spectrum [7]. The discrepancy between those chronic trials and the present synthesis likely reflects factors intrinsic to the acute setting: shorter follow-up windows, the dominance of competing risks during hospitalization, hemodynamic and neurohormonal instability that may attenuate measurable disease-modifying effects, and heterogeneous stabilization criteria across trials.

Within the acute setting, our findings are broadly consistent with the individual contributing trials. The DAPA ACT HF–TIMI 68 trial, the largest study, did not demonstrate a significant reduction in cardiovascular death or worsening heart failure with in-hospital initiation [12]. DICTATE-AHF supported the feasibility and tolerability of dapagliflozin co-administered with intravenous loop diuretic therapy in patients hospitalized with acute heart failure [10], and DAPA-RESIST, in patients with established loop diuretic resistance, found dapagliflozin no more effective than metolazone for decongestion but associated with less biochemical disturbance [11]. Set against trials of other agents, EMPULSE demonstrated improvement in a hierarchical clinical benefit composite at 90 days with empagliflozin in stabilized acute heart failure [2], and SOLOIST-WHF showed reductions in cardiovascular events with sotagliflozin initiated before or shortly after discharge in patients with diabetes and recent worsening heart failure [9]. Differences in eligibility criteria—most importantly stabilization requirements and diabetes status—comparator design, endpoint definitions, and event ascertainment likely contribute to the variation across these programs. Background guideline-directed therapy also varied across the included trials, which spanned an era encompassing increasing uptake of angiotensin receptor–neprilysin inhibitors, and completeness of foundational therapy is a plausible effect modifier.

Diabetes prevalence varied across trials and consistent quantitative reporting at the cohort level was limited; this should be borne in mind when interpreting the metabolic and DKA signals discussed below.

### 4.3. Potential Mechanisms and Clinical Implications

The reduction (non-significant) in body weight observed with dapagliflozin is best interpreted in this context as a predominant marker of enhanced decongestion. Over the short follow-up windows of acute heart failure trials, the dominant contributor to weight change is fluid balance rather than adiposity, and the magnitude observed is consistent with augmented natriuresis and osmotic diuresis when SGLT2 inhibition is added to intravenous loop diuretic therapy [8]. In trials with longer follow-up, however, a contribution from sustained glucosuric caloric loss and modest adipose change cannot be excluded, and the pooled estimate likely reflects a mixed contribution rather than fluid balance alone. Direct decongestion endpoints—including NT-proBNP and BNP trajectories, KCCQ scores, dyspnea visual analogue scales, and hemoconcentration markers—were not synthesized, either because they were not consistently reported across trials or were not prespecified outcomes of this analysis; the decongestion interpretation should therefore be regarded as inferential rather than directly demonstrated. Heart failure hospitalization, the most clinically meaningful post-discharge endpoint is compatible with both a null effect and a clinically meaningful reduction of approximately one third; the included trials were collectively underpowered for this endpoint. A directional but imprecise reduction in length of hospital stay was also observed, which, although non-significant, would be operationally relevant at scale if confirmed in adequately powered trials.

Glucose-independent mechanisms proposed for SGLT2 inhibitors, including reductions in cardiac preload and afterload, attenuation of inflammation and adverse cardiac remodeling, and a postulated shift in myocardial substrate utilization, may operate less efficiently against a background of acute decompensation, neurohormonal activation, and renal stress [8]. SGLT2 inhibitor initiation is also recognized to produce a small, reversible reduction in eGFR within the first weeks of therapy that is hemodynamic in origin and distinct from acute kidney injury [8]; the absence of a significant AKI signal in this synthesis is consistent with that distinction.

For the practising clinician, the practical message is twofold. Starting dapagliflozin during hospitalization for acute heart failure appears to introduce detectable safety across the available randomized evidence, but it is not associated with greater weight reduction. Hypoglycemia events were infrequent and not increased with dapagliflozin, consistent with the established mechanism of SGLT2 inhibition, which does not directly cause hypoglycemia in the absence of concomitant insulin or insulin secretagogue (e.g., sulfonylurea) therapy [8].

### 4.4. Strengths and Limitations

This synthesis has several strengths. It is restricted to randomized evidence on dapagliflozin specifically, incorporates the most recently reported acute heart failure trials, and prespecifies timing of initiation as a subgroup variable. Leave-one-out sensitivity analyses are reported for all outcomes, increasing transparency around the stability of pooled estimates. The protocol was prospectively registered with PROSPERO and the conduct adhered to PRISMA reporting guidelines [14].

Several limitations require explicit acknowledgement. First, ejection fraction inclusion criteria varied across trials and patient-level stratification by ejection fraction was not feasible. Second, follow-up period was generally short, leaving open the possibility that benefits accruing beyond the index hospitalization are not captured.

### 4.5. Conclusions

In this systematic review and meta-analysis of 10 randomized controlled trials, initiation of dapagliflozin during hospitalization in adults with acute heart failure significantly reduced all-cause mortality, but not cardiovascular death, heart failure hospitalization, or length of stay in the all-population analysis. Heart failure hospitalization in the all-population analysis was directionally consistent with the chronic-phase evidence base. The available randomized evidence did not detect a statistically significant safety signal for acute kidney injury, hypotension, or hypoglycemia, although it did for the urinary tract infections; clinical decisions should weigh these uncertainties alongside the absence of demonstrated rehospitalization benefit in the all-population analysis. Adequately powered trials with prospectively prespecified, AHF-specific and timing-stratified analyses are required to determine whether early in-hospital initiation of dapagliflozin offers benefit on hard clinical endpoints in patients hospitalized with acute heart failure.

## Figures and Tables

**Table 1:** Baseline characteristics of included Randomized Controlled Trials.

Study	Year	Trial Identifier	Trial Design	Country	Dapagliflozin Dose	Timing of Dapagliflozin Introduction	Last Follow-Up
Ibrahim et al. [26]	2020	NCT04385589	RCT, 2 arms	Egypt	10 mg once daily	Intra-hospital	1 week
DAPA-RESPONSE-AHF [28]	2023	NCT05406505	RCT, placebo-controlled, double-blind	Egypt	10 mg once daily	Within 24 h of admission	9 weeks
DICTATE-AHF [10]	2024	NCT04298229	RCT, 2 arms, open-label, controlled	USA	10 mg once daily	Within 24 h of hospital presentation	4 weeks
Yeoh et al. [11]	2023	NCT04860011	RCT, 2 arms, open-label	UK	10 mg once daily	Within 24 h of diagnosis of diuretic resistance	28 weeks
Charaya et al. [31,32,33] *	2023	NCT04778787	RCT, 2 arms, open-label	Russia	10 mg once daily	within 12 h after admission	1 week
Mocan et al. [34]	2025	ISRCTN17064177	RCT, 2 arms, open-label	Romania	10 mg once daily	Within 24 h of hospital presentation	4 weeks
DAPA ACT HF-TIMI 68 [12]	2025	NCT04363697	RCT, 2 arms, placebo-controlled, double-blind	USA	10 mg once daily	At least 24 h and no later than 14 days after hospital admission	8 weeks
ROAD-ADHF [35]	2025	UMIN000044342	RCT, 2 arms, open label	Japan	10 mg once daily	within 24 h after admission	1 week
Gojaseni et al. [36]	2024	TCTR20221003002	RCT, 2 arms, open-label	Thailand	10 mg once daily	within 24 h after enrollment	4 weeks
Zhou et al. [37]	2025	NR	RCT, 2 arms, single blind	China	10 mg once daily	within 24 h after enrollment	48 weeks

RCT = Randomized Controlled Trial; NR = Not Reported. * Trial publication linkage table for Charaya et al. [31,32,33] (NCT04778787) is provided in Supplementary Table S4.

**Table 2:** Baseline characteristics of included participants.

Study	Year	Participants (*n*)			Mean Age (Years)		Males *n* (%)		Females *n* (%)		Diabetes Type 2, %		Hypertensive, *n*, %		History of Ischemic Heart Disease %		ACEi, ARBs *n* (%)		LVEF, %		Mean eGFR (ml/min/1.73 m^2^)		Diuretics (%)		BMI		HbA1c %	
		Dapagliflozin	Control	Total	Dapagliflozin	Control	Dapagliflozin	Control	Dapagliflozin	Control	Dapagliflozin	Control	Dapagliflozin	Control	Dapagliflozin	Control	Dapagliflozin	Control	Dapagliflozin	Control	Dapagliflozin	Control	Dapagliflozin	Control	Dapagliflozin	Control	Dapagliflozin	Control
Ibrahim et al. [26]	2020	50	50	100	62.02 ± 8.81	60.64 ± 9.90	28 (56%)	26 (52%)	22 (44%)	24 (48%)	50 (100%)	50 (100%)	28 (56%)	31 (62%)	NR	NR	46	45	32.54 ± 2.99	32.23 ± 2.49	>45	>45	5 (10%)	4 (8%)	27.78 ± 2.33	28.23 ± 3.32	8.61 ± 1.2	9.09 ± 2.1
DAPA-RESPONSE-AHF [28]	2023	45	42	87	61.1 ± 11.8	63.9 ± 10	35 (77.8%)	27 (64.3%)	10 (22.2%)	15 (35.7%)	16 (35.6%)	22 (52.4%)	22 (48.9%)	21 (50%)	8 (17.8%)	12 (28.6%)	21 (46.7%)	16 (38.1%)	34	36.5	>30	>30	38 (84.4%)	37 (88%)	NR	NR	NR	NR
DICTATE-AHF [10]	2024	119	119	238	65 (56–73) * Mean: 64.6 ± 12.8	64 (55–74) * Mean: 64.3 ± 14.3	78 (66%)	67 (56%)	41 (34%)	52 (44%)	84 (71%)	85 (71%)	107 (90%)	96 (81%)	102 (86%)	103 (87%)	59 (50%)	64 (54%)	45 (26–56) *	35 (25–60) *	51 (43–68) *	54 (40–71) *	55 (46%)	66 (55%)	32 (28–39) * Mean: 33 ± 8.3	34 (28–42) * Mean: 34.7 ± 10.5	6.7 (6.0–8.1) * Mean: 6.9 ± 1.6	6.7 (6.0–7.9) * Mean: 6.9 ± 1.4
Yeoh et al. [11]	2023	30	31	61	79 (73–86) * Mean: 79.4 ± 10.1	79 (68–84) * Mean: 76.8 ± 12.4	13 (43%)	15 (48%)	17 (57%)	16 (52%)	19 (63%)	9 (29%)	NR	NR	9 (30%)	12 (39%)	7 (23)	12 (40)	45 (35–55) *	45 (35–55) *	40.7 (34.1–50.7)	40.7 (29.2–59.1)	26 (87%)	28 (90%)	32 (27–36) * Mean: 31.6 ± 7	33 (28–38) * Mean: 33 ± 7.77	6.20 ± 1.27	5.80 ± 0.81
Charaya et al. [31,32,33]	2023	140	145	285	72 ± 12	75 ± 13	78 (56%)	73 (50.5%)	62 (44%)	72 (49.5%)	44(31%)	56(38%)	120 (86%)	126 (87%)	73 (52%)	67 (46%)	NR	NR	NR	NR	55.6 ± 20	52 ± 19	NR	NR	NR	NR	NR	NR
Mocan et al. [34]	2025	49	49	98	63.63 ± 10.95	65.31 ± 10.82	40 (81.6%)	42 (85.7%)	9 (18.4%)	7 (14.3%)	10 (20.4%)	15 (30.6%)	NR	NR	14 (28.6%)	10 (20.4%)	NR	NR	26.03 ± 8.81	28.13 ± 9.75	70.35 ± 20.52	73.98 ± 20.05	-	-	Male: 28.49 ± 5.65 Female: 27.39 ± 5.08 Cumulative mean: 28.29 ± 5.56	Male: 29.12 ± 9.03 Female: 23.73 ± 2.67 Cumulative mean: 28.35 ± 8.49	6.52 ± 1.47	6.02 ± 0.8
DAPA ACT HF-TIMI 68 [12]	2025	1218	1183	2401	69 (58–77) * Mean: 68.0 ± 14.1	68 (58–76) * Mean: 67.3 ± 13.4	815 (66.9%)	771 (65.2%)	403 (33.1%)	412 (34.8%)	437 (35.9%)	415 (35.1%)	943 (77.4%)	935 (79.0%)	243/1196 (20.3%)	222/1167 (19.0%)	539 (44.3%)	533 (45.1%)	30.0 (20.0–45.0) *	30.0 (21.0–45.0) *	63.5 (48.5–83.0) *	62.5 (47.2–80.7) *	1041 (85.5%)	998 (84.4%)	29.0 (24.9–34.7) * Mean: 29.6 ± 7.3	29.5 (25.4–35.4) * Mean: 30.1 ± 7.4	NR	NR
ROAD-ADHF [35]	2025	56	58	114	74 [63, 85] * Mean: 74 ± 16.74	78 [70, 85] * Mean: 77.7 ± 11.4	35 (62.5%)	39 (67.2%)	21 (37.5%)	19 (32.8%)	15 (26.8%)	9 (15.5%)	34 (60.7%)	36 (62.1%)	23 (43.3%)	23 (41.8%)	17 (30.4%)	13 (22.4%)	35 [29, 39] *	30 [25, 36] *	NR	NR	8 (14.3%)	6 (10.3%)	24.0 [19.9, 27.5] * Mean: 23.8 ± 5.8	23.6 [21.7, 26.1] * Mean: 23.8 ± 3.3	6.2 [5.8, 6.6] * Mean:6.2 ± 0.6	5.9 [5.7, 6.3] * Mean: 6.0 ± 0.46
Gojaseni et al. [36]	2024	12	13	25	67.0 ± 17.4	67.2 ± 13.4	7 (58.3%)	7 (53.8%)	5 (41.7%)	6 (46.2%)	5 (41.7%)	4 (30.8%)	9 (75.0%)	8 (61.5%)	1 (8.3%)	4 (30.8%)	3 (25.0%)	7 (53.8%)	42.4 ± 17.0	41.8 ± 16.7	61.3 ± 20.7	64.9 ± 24.4	4 (33.3%)	4 (30.8%)	NR	NR	NR	NR
Zhou et al. [37]	2025	50	48	98	65.27 ± 11.87	68.05 ± 9.79	30 (60%)	28 (58.33%)	20 (40%)	20 (41.67%)	25 (50%)	30 (62.5%)	37 (74%)	38 (79.17%)	-	-	-	-	49.00 ± 9.37	48.33 ± 10.22	-	-	-	-	23.50 ± 2.70	23.66 ± 2.57	7.10 ± 1.66	6.52 ± 1.35

* Median (IQR); ACEi = angiotensin-converting enzyme inhibitors; ARBs = angiotensin receptor blockers; BMI = body mass index; CONTROL = control group; eGFR = estimated glomerular filtration rate; HbA1c = glycated hemoglobin; LVEF = left ventricular ejection fraction; n = number of participants; NR = Not Reported.

## Data Availability

Data available from corresponding author on request.

## Supplementary Materials

The following supporting information can be downloaded at https://www.mdpi.com/article/10.3390/biomedicines14102319/s1, Table S1: PRISMA 2020 checklist; Table S2: search strategy and databases used for literature selection; Table S3: GRADE certainty of evidence assessment; Table S4: trial publication linkage table for Charaya et al. (NCT04778787) [31,32,33]; Figure S1: risk-of-bias summary plot for the included randomized controlled trials; Figure S2: risk-of-bias traffic light plot for the included randomized controlled trials; Figures S3–S24: supplementary forest plots and sensitivity analyses; Figures S25 and S26: subgroup analyses according to the 12 h dapagliflozin initiation threshold.
