# Supplementary material for: Early Initiation of Dapagliflozin in Acute Heart Failure: A Systematic Review and Meta-Analysis of Randomized Controlled Trials
Source: Biomedicines. 2026 Oct 13;14(10):2319. doi: 10.3390/biomedicines14102319 (PMC13701616; doi:10.3390/biomedicines14102319)
Supplement: Supplementary file 1 [file biomedicines-14-02319-s001.zip › biomedicines-4506888-supplementary.pdf]

Supplementary Materials

Early Initiation of Dapagliflozin in Acute Heart Failure: A Systematic Review and Meta-Analysis of Randomized Controlled Trials

This file includes Supplementary Tables S1-S4 and Supplementary Figures S1-S26.

Supplementary Table S1. PRISMA 2020 checklist

Supplementary Table S2. Search strategy and databases used for literature selection.

Supplementary Table S3. GRADE Certainty of Evidence Assessment

Supplementary Table S4. Trial publication linkage table for Charaya et al. (NCT04778787)

Supplementary Figure S1. Risk-of-bias summary plot for the included randomized controlled trials.

Supplementary Figure S2. Risk-of-bias traffic light plot for the included randomized controlled trials.

Supplementary Figure S3. Forest plot for cardiovascular death.

Supplementary Figure S4. Sensitivity analysis for all-cause mortality.

Supplementary Figure S5. Forest plot for stroke.

Supplementary Figure S6. Forest plot for heart failure hospitalization/worsening heart failure.

Supplementary Figure S7. Leave-one-out sensitivity analysis for heart failure hospitalization/worsening heart failure.

Supplementary Figure S8. Forest plot for all-cause readmission.

Supplementary Figure S9. Sensitivity analysis for all-cause readmission. (zhou et al. 2025 removed)

Supplementary Figure S10. Forest plot for length of hospital stay

Supplementary Figure S11. Leave-one-out sensitivity analysis for length of hospital stay.

Supplementary Figure S12. Forest plot for weight/BMI change.

Supplementary Figure S13. Leave-one-out sensitivity analysis for weight/BMI change.

Supplementary Figure S14. Forest plot for eGFR change.

Supplementary Figure S15. Leave-one-out sensitivity analysis for eGFR change.

Supplementary Figure S16. Forest plot for serum creatinine change.

Supplementary Figure S17. Forest plot for acute kidney injury.

Supplementary Figure S18. Leave-one-out sensitivity analysis for acute kidney injury.

Supplementary Figure S19. Forest plot for urinary tract infection. ($p=0.009$)

Supplementary Figure S20. Leave-one-out sensitivity analysis for urinary tract infection.

Supplementary Figure S21. Forest plot for hypoglycemia.

Supplementary Figure S22. Leave-one-out sensitivity analysis for hypoglycemia

Supplementary Figure S23. Forest plot for hypotension.

Supplementary Figure S24. Leave-one-out sensitivity analysis for hypotension.

Supplementary Figure S25. Subgroup analysis of length of hospital stay according to the 12-hour dapagliflozin initiation threshold.

Supplementary Figure S26. Subgroup analysis of weight/BMI change according to the 12-hour dapagliflozin initiation threshold.

Supplementary Table S1. PRISMA 2020 checklist

Section and Topic	Item #	Checklist item	Location where item is reported
**TITLE**			
Title	1	Identify the report as a systematic review.	Page 1
**ABSTRACT**			
Abstract	2	See the PRISMA 2020 for Abstracts checklist.	Page 1,2
**INTRODUCTION**			
Rationale	3	Describe the rationale for the review in the context of existing knowledge.	Page 3,4
Objectives	4	Provide an explicit statement of the objective(s) or question(s) the review addresses.	Page 3,4
**METHODS**			
Eligibility criteria	5	Specify the inclusion and exclusion criteria for the review and how studies were grouped for the syntheses.	Page 4
Information sources	6	Specify all databases, registers, websites, organisations, reference lists and other sources searched or consulted to identify studies. Specify the date when each source was last searched or consulted.	Page 5
Search strategy	7	Present the full search strategies for all databases, registers and websites, including any filters and limits used.	Page 4, Supplementary material
Selection process	8	Specify the methods used to decide whether a study met the inclusion criteria of the review, including how many reviewers screened each record and each report retrieved, whether they worked independently, and if applicable, details of automation tools used in the process.	Page 4
Data collection process	9	Specify the methods used to collect data from reports, including how many reviewers collected data from each report, whether they worked independently, any processes for obtaining or confirming data from study investigators, and if applicable, details of automation tools used in the process.	Page 4
Data items	10a	List and define all outcomes for which data were sought. Specify whether all results that were compatible with each outcome domain in each study were sought (e.g. for all measures, time points, analyses), and if not, the methods used to decide which results to collect.	Page 5
	10b	List and define all other variables for which data were sought (e.g. participant and intervention characteristics, funding sources). Describe any assumptions made about any missing or unclear information.	Page 5
Study risk of bias assessment	11	Specify the methods used to assess risk of bias in the included studies, including details of the tool(s) used, how many reviewers assessed each study and whether they worked independently, and if applicable, details of automation tools used in the process.	Page 5
Effect measures	12	Specify for each outcome the effect measure(s) (e.g. risk ratio, mean difference) used in the synthesis or presentation of results.	Page 4
Synthesis methods	13a	Describe the processes used to decide which studies were eligible for each synthesis (e.g. tabulating the study intervention characteristics and comparing against the planned groups for each synthesis (item #5)).	Page 4
	13b	Describe any methods required to prepare the data for presentation or synthesis, such as handling of missing summary statistics, or data conversions.	-
	13c	Describe any methods used to tabulate or visually display results of individual studies and syntheses.	-

Section and Topic	Item #	Checklist item	Location where item is reported
	13d	Describe any methods used to synthesize results and provide a rationale for the choice(s). If meta-analysis was performed, describe the model(s), method(s) to identify the presence and extent of statistical heterogeneity, and software package(s) used.	-
	13e	Describe any methods used to explore possible causes of heterogeneity among study results (e.g. subgroup analysis, meta-regression).	-
	13f	Describe any sensitivity analyses conducted to assess robustness of the synthesized results.	-
Reporting bias assessment	14	Describe any methods used to assess risk of bias due to missing results in a synthesis (arising from reporting biases).	Page 5
Certainty assessment	15	Describe any methods used to assess certainty (or confidence) in the body of evidence for an outcome.	Page 5
**RESULTS**			
Study selection	16a	Describe the results of the search and selection process, from the number of records identified in the search to the number of studies included in the review, ideally using a flow diagram.	Page 6-9
	16b	Cite studies that might appear to meet the inclusion criteria, but which were excluded, and explain why they were excluded.	Page 6-9
Study characteristics	17	Cite each included study and present its characteristics.	Page 6-9
Risk of bias in studies	18	Present assessments of risk of bias for each included study.	Page 6-9
Results of individual studies	19	For all outcomes, present, for each study: (a) summary statistics for each group (where appropriate) and (b) an effect estimate and its precision (e.g. confidence/credible interval), ideally using structured tables or plots.	Page 6-9
Results of syntheses	20a	For each synthesis, briefly summarise the characteristics and risk of bias among contributing studies.	Page 6-9
	20b	Present results of all statistical syntheses conducted. If meta-analysis was done, present for each the summary estimate and its precision (e.g. confidence/credible interval) and measures of statistical heterogeneity. If comparing groups, describe the direction of the effect.	Page 6-9
	20c	Present results of all investigations of possible causes of heterogeneity among study results.	Page 6-9
	20d	Present results of all sensitivity analyses conducted to assess the robustness of the synthesized results.	Page 6-9
Reporting biases	21	Present assessments of risk of bias due to missing results (arising from reporting biases) for each synthesis assessed.	Page 6-9
Certainty of evidence	22	Present assessments of certainty (or confidence) in the body of evidence for each outcome assessed.	Page 6-9
**DISCUSSION**			
Discussion	23a	Provide a general interpretation of the results in the context of other evidence.	Page 12
	23b	Discuss any limitations of the evidence included in the review.	Page 12
	23c	Discuss any limitations of the review processes used.	Page 12
	23d	Discuss implications of the results for practice, policy, and future research.	Page 13
**OTHER INFORMATION**			
Registration and protocol	24a	Provide registration information for the review, including register name and registration number, or state that the review was not registered.	Page 3,4

Section and Topic	Item #	Checklist item	Location where item is reported
	24b	Indicate where the review protocol can be accessed, or state that a protocol was not prepared.	Page 3,4
	24c	Describe and explain any amendments to information provided at registration or in the protocol.	Page 3,4
Support	25	Describe sources of financial or non-financial support for the review, and the role of the funders or sponsors in the review.	-
Competing interests	26	Declare any competing interests of review authors.	Page 14
Availability of data, code and other materials	27	Report which of the following are publicly available and where they can be found: template data collection forms; data extracted from included studies; data used for all analyses; analytic code; any other materials used in the review.	Page 14

Supplementary Table S2. Search strategy and databases used for literature selection.

Database	Search Strategy	# of Results
PubMed	((((((((((((((Heart Failure[MeSH Terms]) OR (Heart Failure, Diastolic[MeSH Terms])) OR (Heart Failure, Systolic[MeSH Terms])) OR (Cardiac Failure)) OR (Heart Decompensation)) OR (Heart Failure, Right Sided)) OR (Heart Failure, Left-Sided)) OR (Left-Sided Heart Failure)) OR (Acute Heart Failure)) OR (Acute Cardiac Failure)) OR (Acute heart decompensation)) AND (dapagliflozin[Supplementary Concept])) OR ((2S,3R,4R,5S,6R)-2-(4-chloro-3-(4-ethoxybenzyl)phenyl)-6- (hydroxymethyl)tetrahydro-2H-pyran-3,4,5-triol)) OR (2-(3-(4-ethoxybenzyl)-4-chlorophenyl)-6-hydroxymethyltetrahydro-2H-pyran-3,4,5-triol)) OR (BMS 512148)) OR (BMS-512148)) OR (Forxiga) AND (clinicaltrial[Filter] OR randomizedcontrolledtrial[Filter])	572
Scopus	(dapagliflozin OR Forxiga OR BMS-512148) AND ("ejection fraction" OR "myocardial infarction" OR "ventricular dysfunction" OR "acute coronary syndrome" OR STEMI)	903
Embase	(acute heart failure OR ACS OR Acute Cardiac Failure OR Acute heart decompensation) AND (dapagliflozin OR PCI OR Forxiga OR BMS-512148)	1176
Cochrane CENTRAL	*Set#1*<br><br>Acute Heart Failure OR Acute Cardiac Failure OR Acute heart decompensation<br>*Set#2*<br><br>dapagliflozin OR Forxiga<br>*Set#3*	140

	Control OR Placebo<br>*Set#4*<br>#1 AND #2 AND #3 AND #4	

Date of Search: 26/1/2026

Supplementary Table S3. GRADE Certainty of Evidence Assessment

Certainty assessment							№ of patients		Effect		Certainty
№ of studies	Study design	Risk of bias	Inconsistency	Indirectness	Imprecision	Other considerations	Dapagliflozin	Control	Relative (95% CI)	Absolute (95% CI)	
**Major Adverse Cardiovascular Event**											
2	randomised trials	not serious	not serious	serious	not serious	none	7/167 (4.2%)	19/164 (11.6%)	**RR 0.36** (0.00 to 37.44)	**74 fewer per 1,000** (from -- to 1,000 more)	⊕⊕⊕◯ Moderate
**Cardiovascular Death**											
2	randomised trials	not serious	not serious	not serious	serious[a]	none	31/1336 (2.3%)	41/1158 (3.5%)	**RR 0.73** (0.02 to 29.49)	**10 fewer per 1,000** (from 35 fewer to 1,000 more)	⊕⊕⊕◯ Moderate[a]
**All-Cause Mortality**											
9	randomised trials	not serious	not serious	not serious	serious[a]	none	56/1710 (3.3%)	85/1588 (5.4%)	**RR 0.67** (0.52 to 0.86)	**18 fewer per 1,000** (from 26 fewer to 7 fewer)	⊕⊕⊕◯ Moderate[a]
**Stroke**											

Certainty assessment							№ of patients		Effect		Certainty
№ of studies	Study design	Risk of bias	Inconsistency	Indirectness	Imprecision	Other considerations	Dapagliflozin	Control	Relative (95% CI)	Absolute (95% CI)	
2	randomised trials	not serious	not serious	not serious	not serious	none	1/168 (0.6%)	2/164 (1.2%)	**RR 0.61** (0.00 to 703.30)	**5 fewer per 1,000** (from -- to 1,000 more)	⊕⊕⊕⊕ High
**Heart Failure Hospitalization**											
4	randomised trials	serious[b]	not serious	not serious	not serious	none	113/1273 (8.9%)	127/1377 (9.2%)	**RR 0.87** (0.59 to 1.29)	**12 fewer per 1,000** (from 38 fewer to 27 more)	⊕⊕⊕◯ Moderate[b]
**Readmission**											
7	randomised trials	not serious	not serious	not serious	serious[a]	none	163/1602 (10.2%)	199/1570 (12.7%)	**RR 0.89** (0.75 to 1.07)	**14 fewer per 1,000** (from 32 fewer to 9 more)	⊕⊕⊕◯ Moderate[a]
**Length of Hospital Stay**											
8	randomised trials	serious[b]	serious[c]	not serious	not serious	none	500	503	-	MD **0.45 Days lower** (0.96 lower to 0.06 higher)	⊕⊕◯◯ Low[b,c]
**eGFR Change**											
4	randomised trials	not serious	very serious[d]	not serious	not serious	none	229	241	-	MD **1.93 ml/min lower** (11.19 lower to 7.34 higher)	⊕⊕◯◯ Low[d]
**Serum Creatinine**											
2	randomised trials	not serious	very serious[d]	not serious	very serious[a]	none	99	97	-	MD **1.71 mg/dl lower** (24.96 lower to 21.54 higher)	⊕◯◯◯ Very low[a,d]

Certainty assessment							№ of patients		Effect		Certainty
№ of studies	Study design	Risk of bias	Inconsistency	Indirectness	Imprecision	Other considerations	Dapagliflozin	Control	Relative (95% CI)	Absolute (95% CI)	
**Weight Change**											
6	randomised trials	not serious	very serious[d]	not serious	not serious	none	444	454	-	MD **0.59 kg lower** (1.43 lower to 0.26 higher)	⨁⨁◯◯ Low[d]

CI: confidence interval; **MD:** mean difference; **RR:** risk ratio

Explanations

a. Variation in Effect

b. Deviation from intended intervention

c. Moderate Hetrogeneity

d. Very High Hetrogeneity

Supplementary Table S4. Trial publication linkage table for Charaya et al. (NCT04778787)

Trial ID (registry #)	Report (citation)	Journal, year	n reported	Outcome(s) reported	Enrollment window	Relationship to other reports
NCT04778787	Charaya et al., *Open Heart* 2022	Open Heart, 2022	102	eGFR/renal function deterioration	earliest cut	Pilot; design paper for the trial
NCT04778787	Charaya et al., *Kardiologiia* 2023	Kardiologiia, 2023	200	AKI (primary), diuretic dose	interim cut	Same cohort, larger n than pilot
NCT04778787	Charaya et al., *Cardiorenal Med* 2023	Cardiorenal Med, 2023	285	Serum sodium change	final/largest cut	Explicitly cites Open Heart pilot as the parent trial

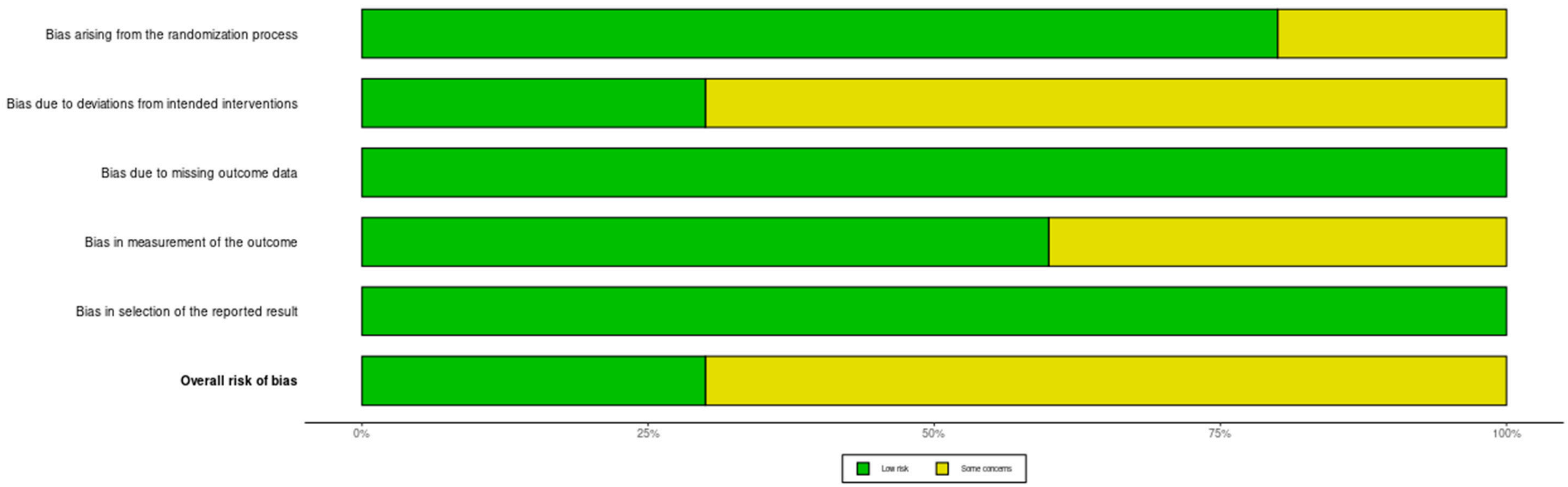

Supplementary Figure S1. Risk-of-bias summary plot for the included randomized controlled trials.

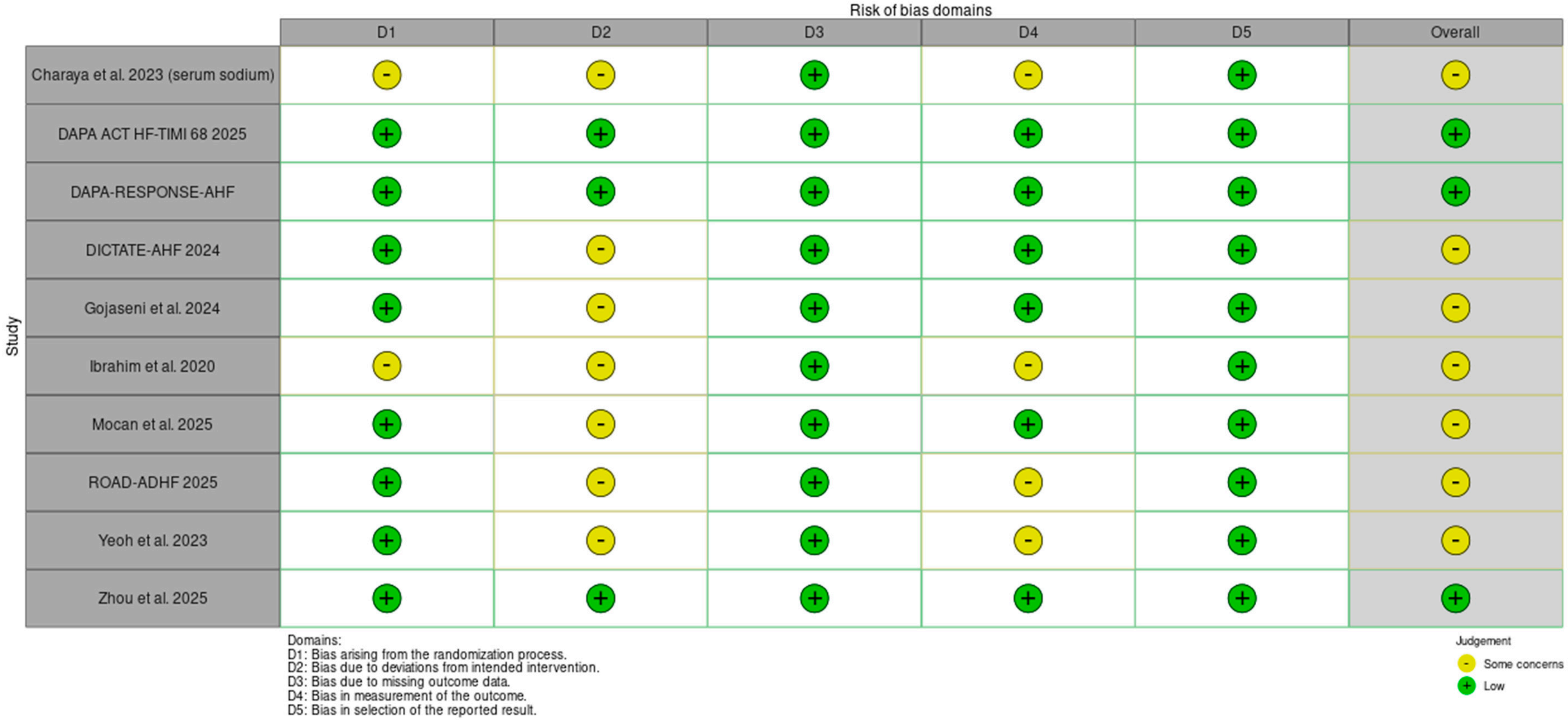

Supplementary Figure S2. Risk-of-bias traffic light plot for the included randomized controlled trials.

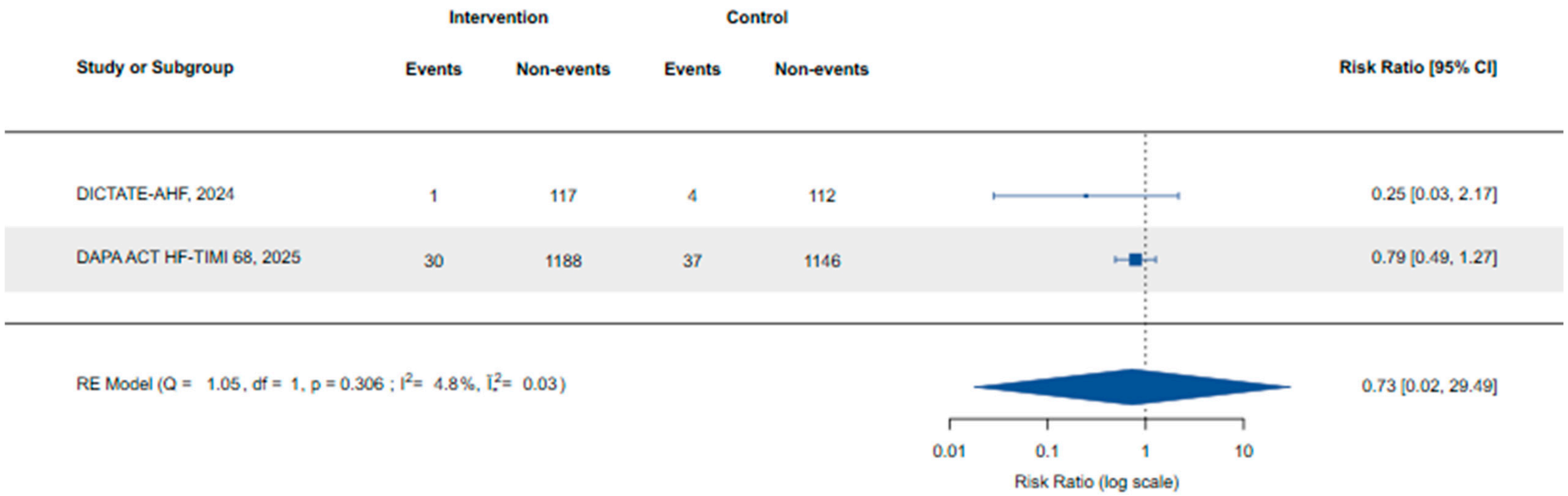

Supplementary Figure S3. Forest plot for cardiovascular death.

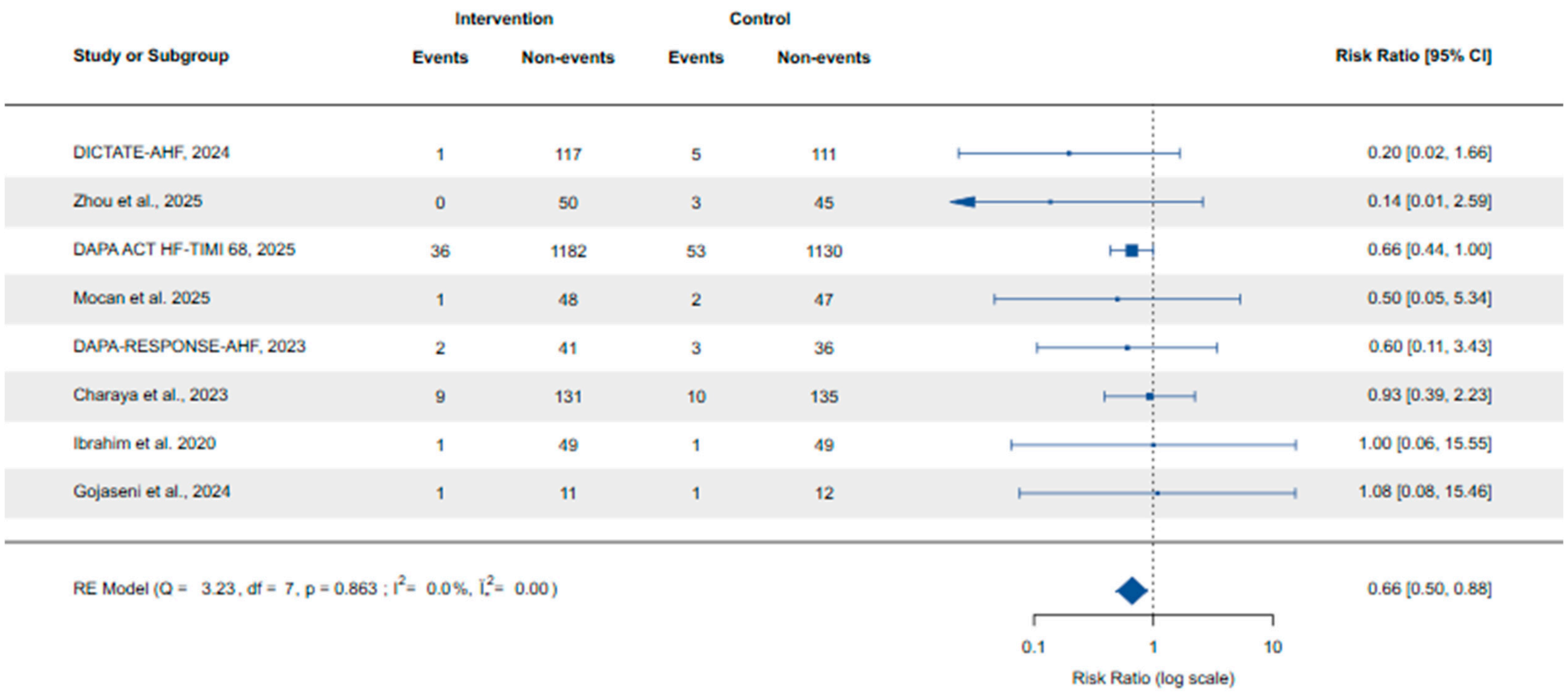

Supplementary Figure S4. Sensitivity analysis for all-cause mortality.

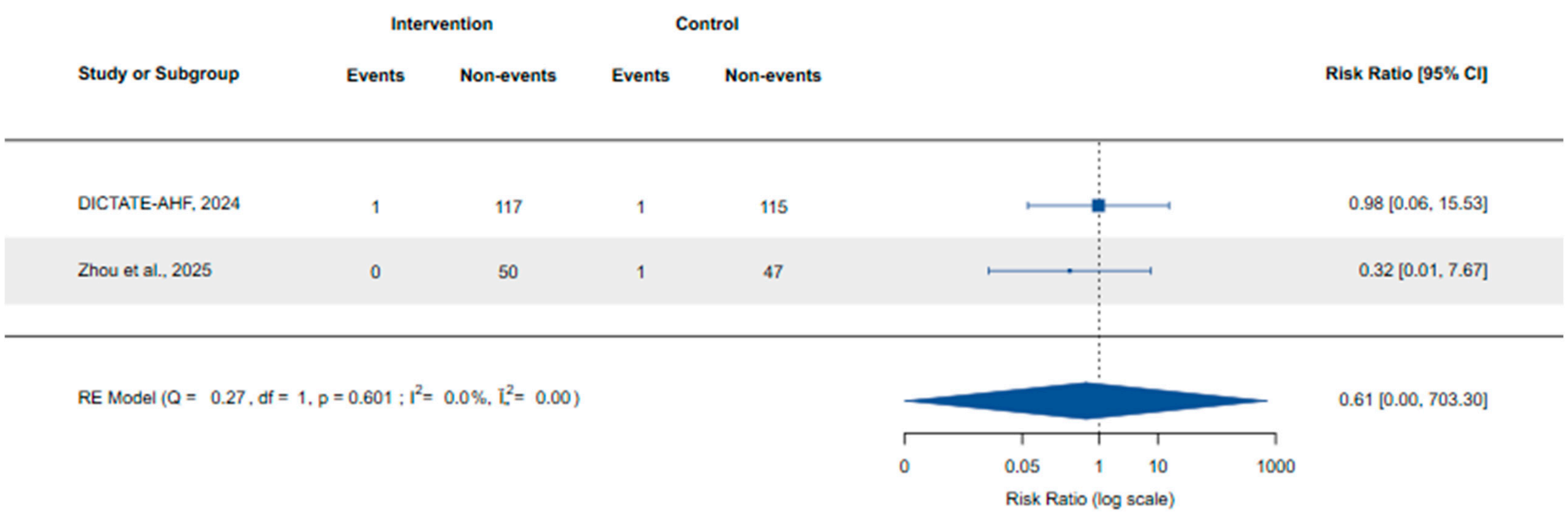

Supplementary Figure S5. Forest plot for stroke.

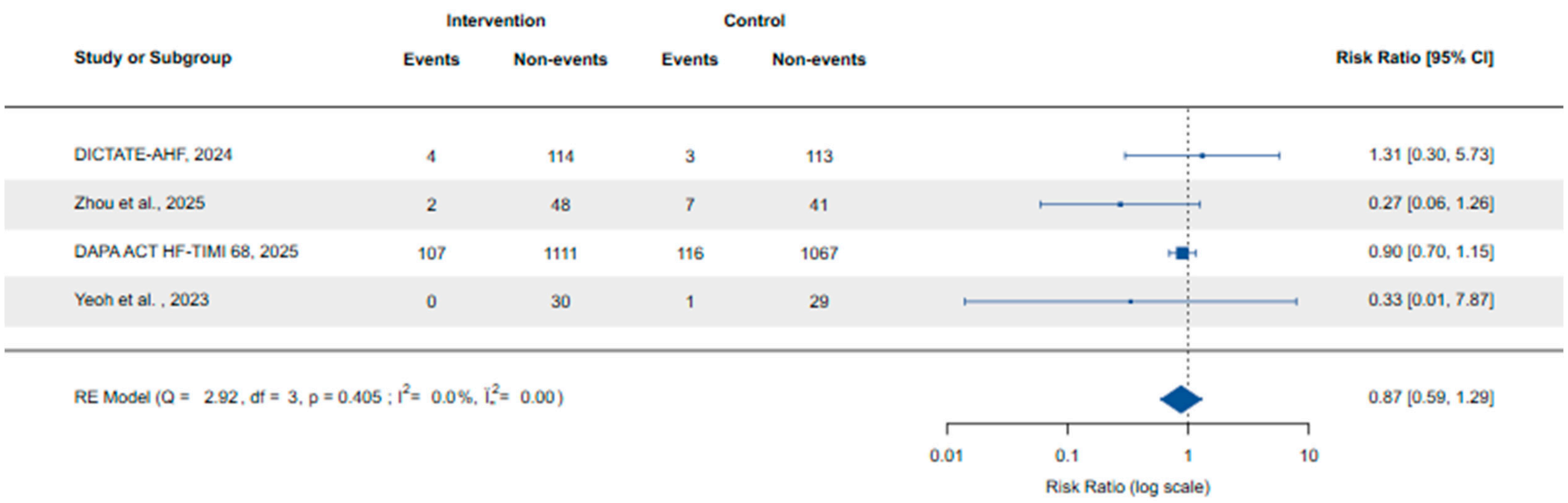

Supplementary Figure S6. Forest plot for heart failure hospitalization/worsening heart failure.

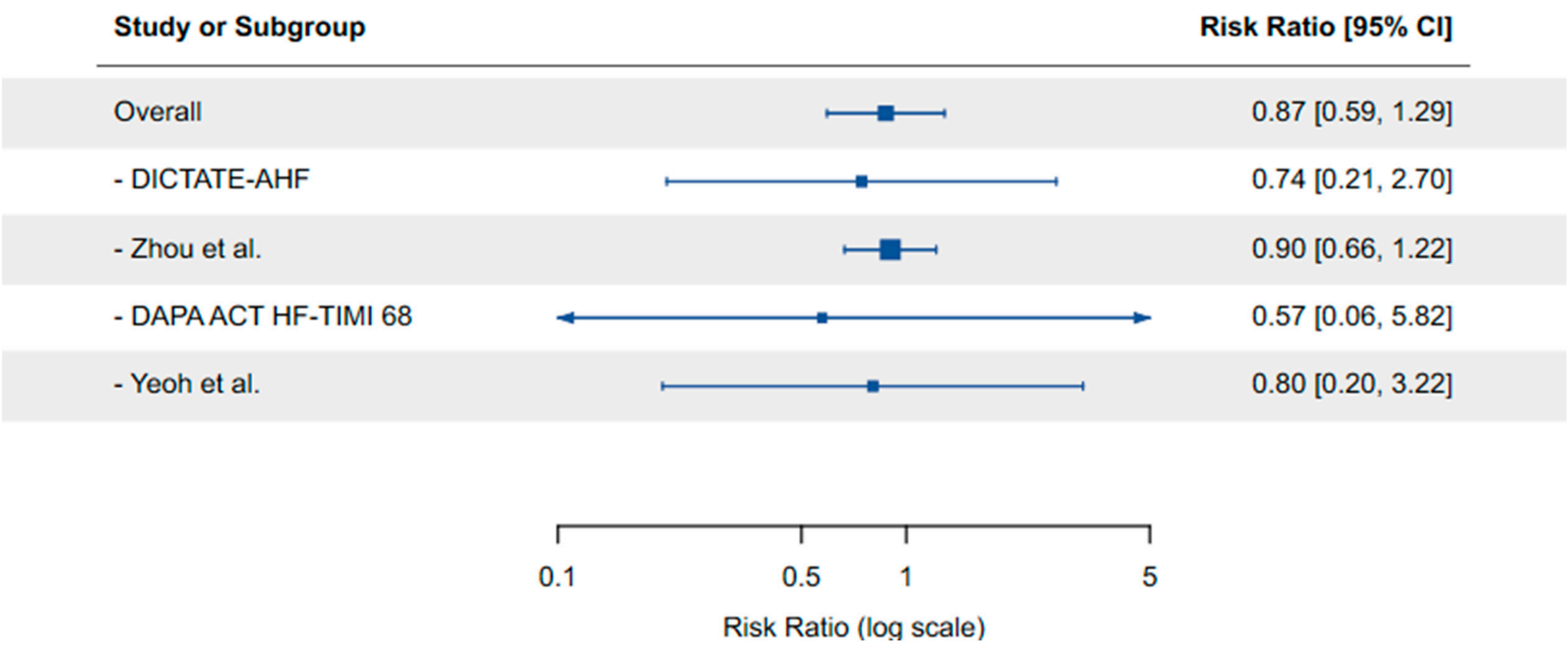

Supplementary Figure S7. Leave-one-out sensitivity analysis for heart failure hospitalization/worsening heart failure.

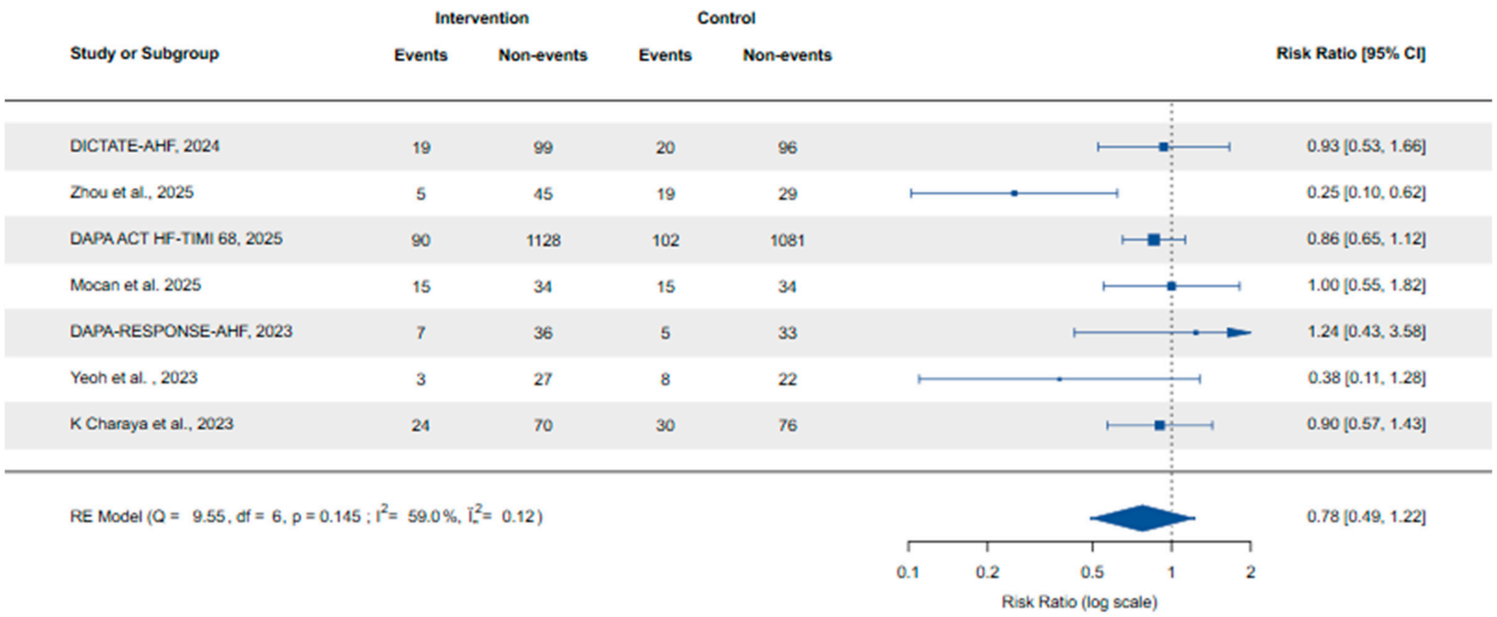

Supplementary Figure S8. Forest plot for all-cause readmission.

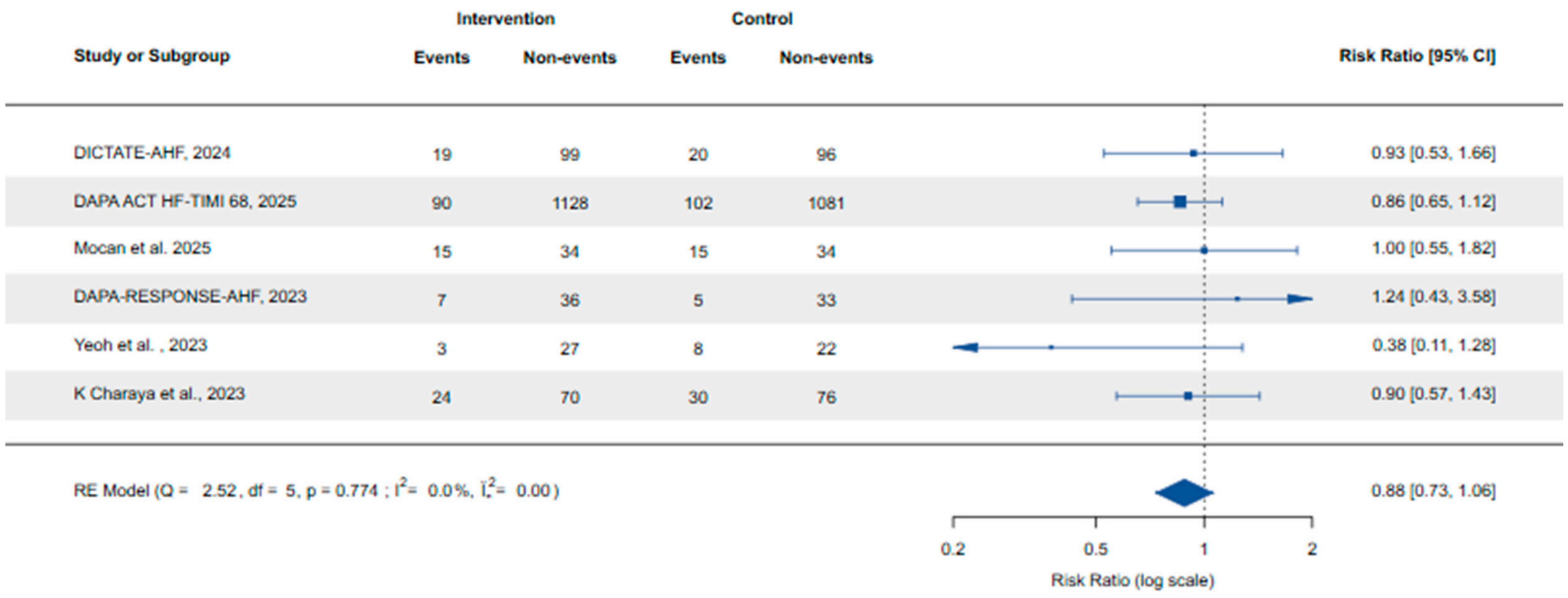

Supplementary Figure S9. Sensitivity analysis for all-cause readmission. (zhou et al. 2025 removed)

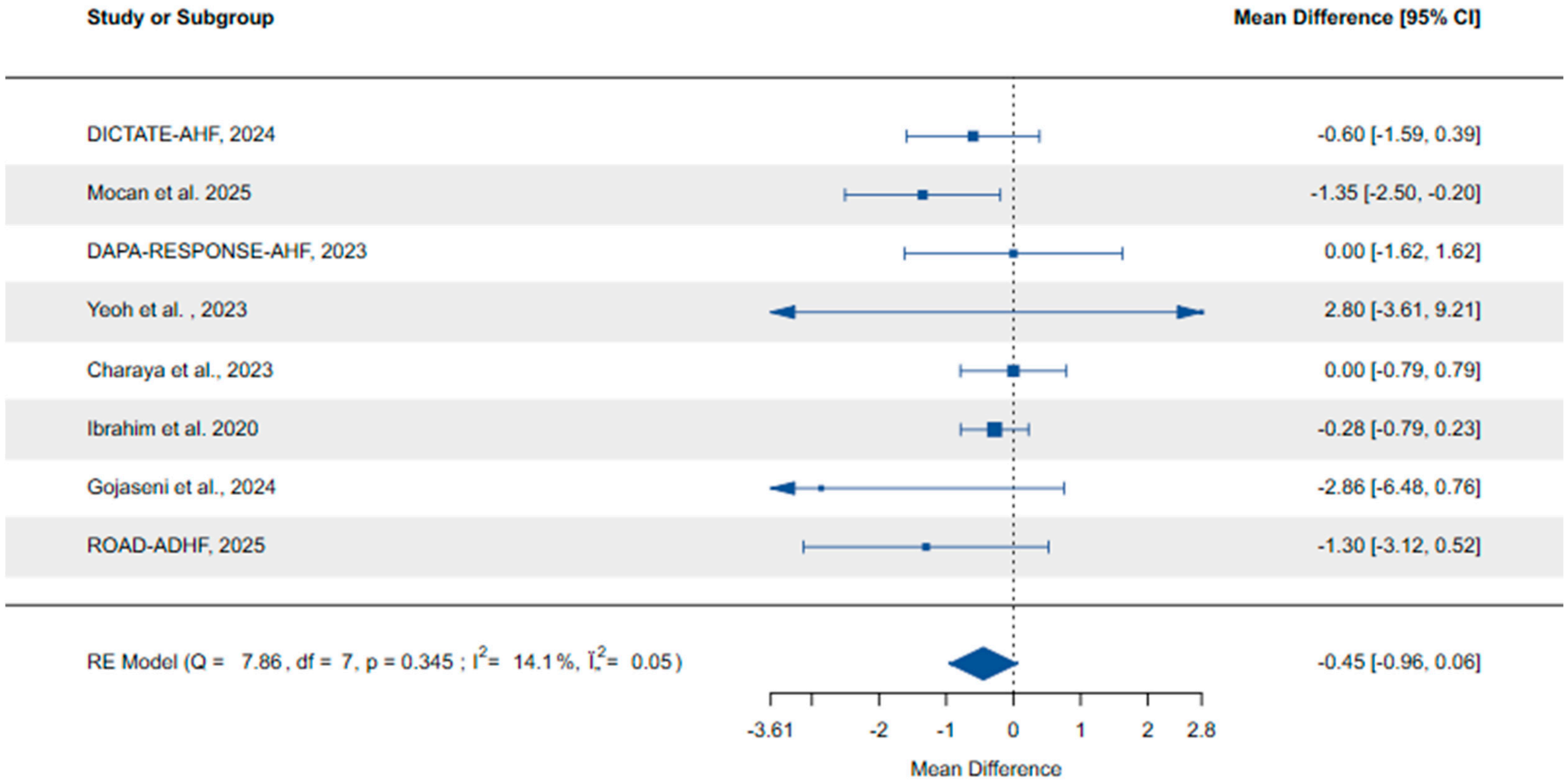

Supplementary Figure S10. Forest plot for length of hospital stay

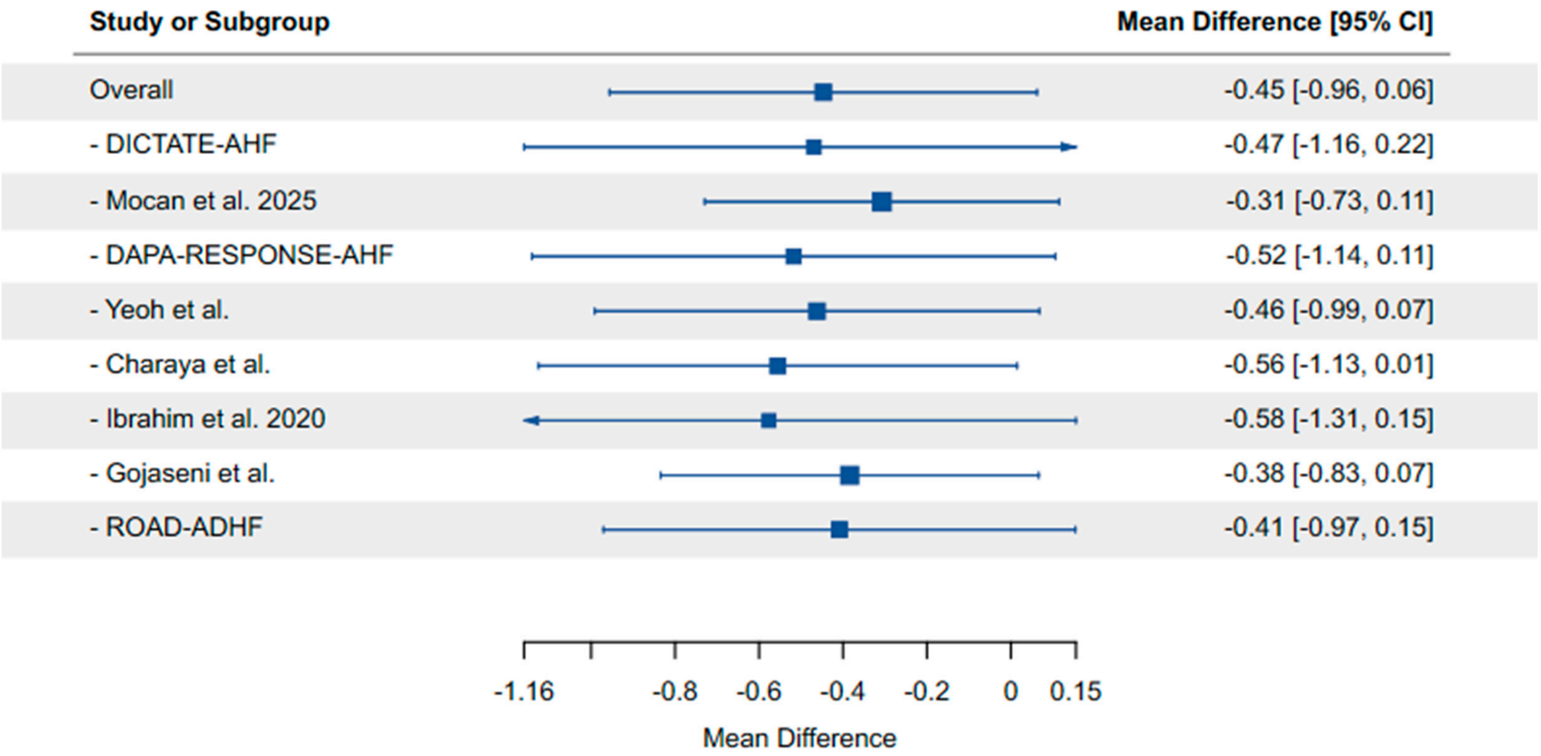

Supplementary Figure S11. Leave-one-out sensitivity analysis for length of hospital stay.

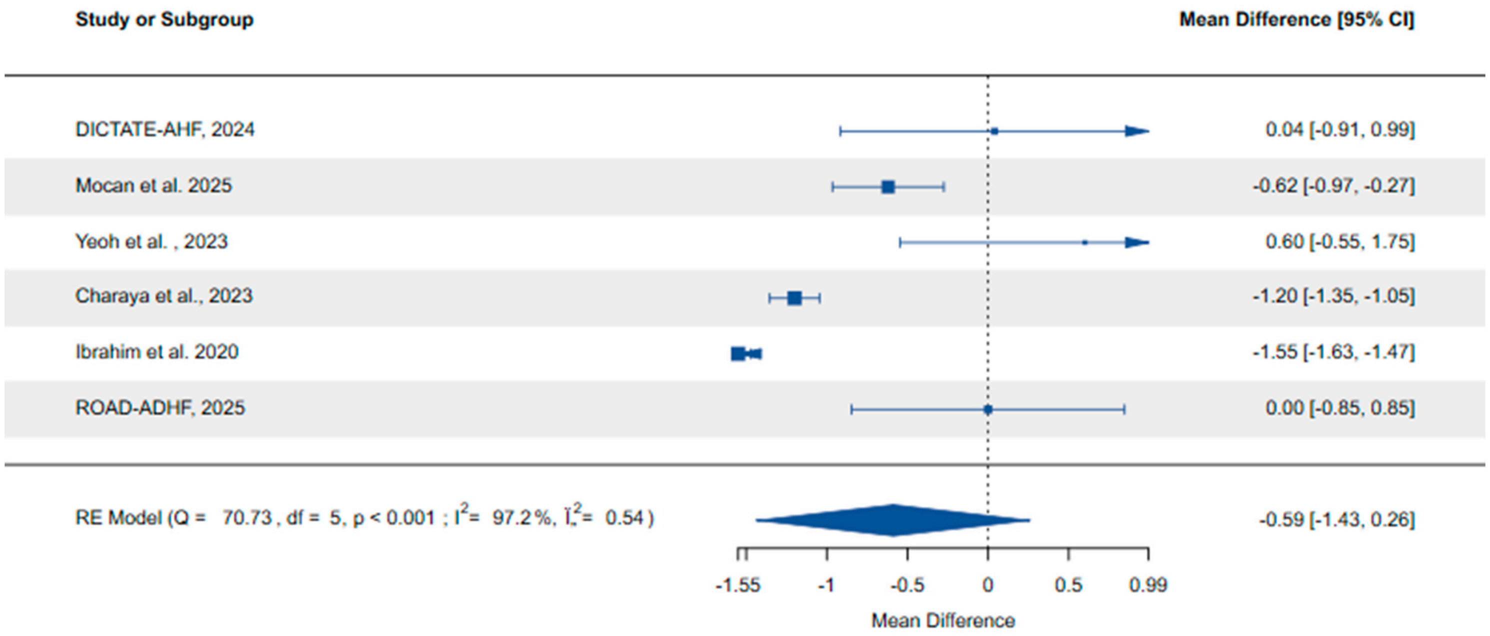

Supplementary Figure S12. Forest plot for weight/BMI change.

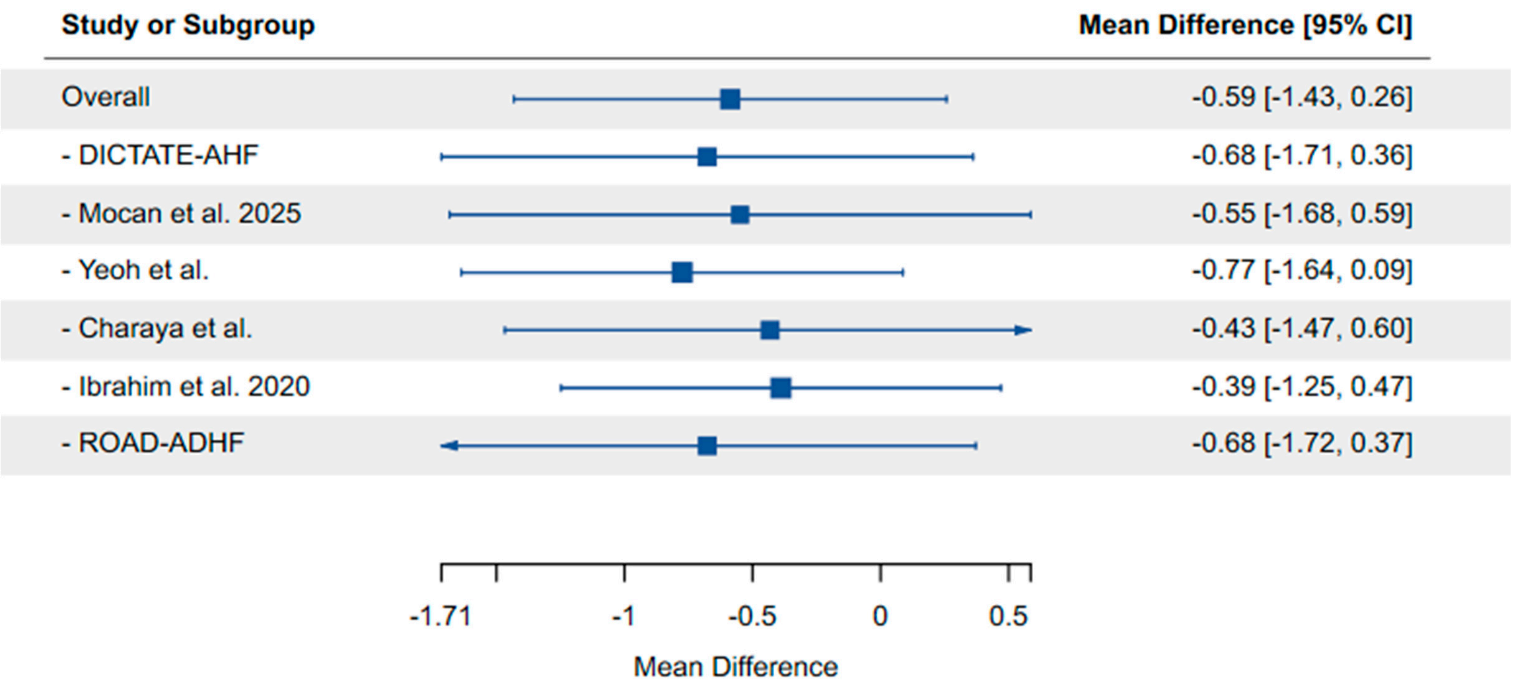

Supplementary Figure S13. Leave-one-out sensitivity analysis for weight/BMI change.

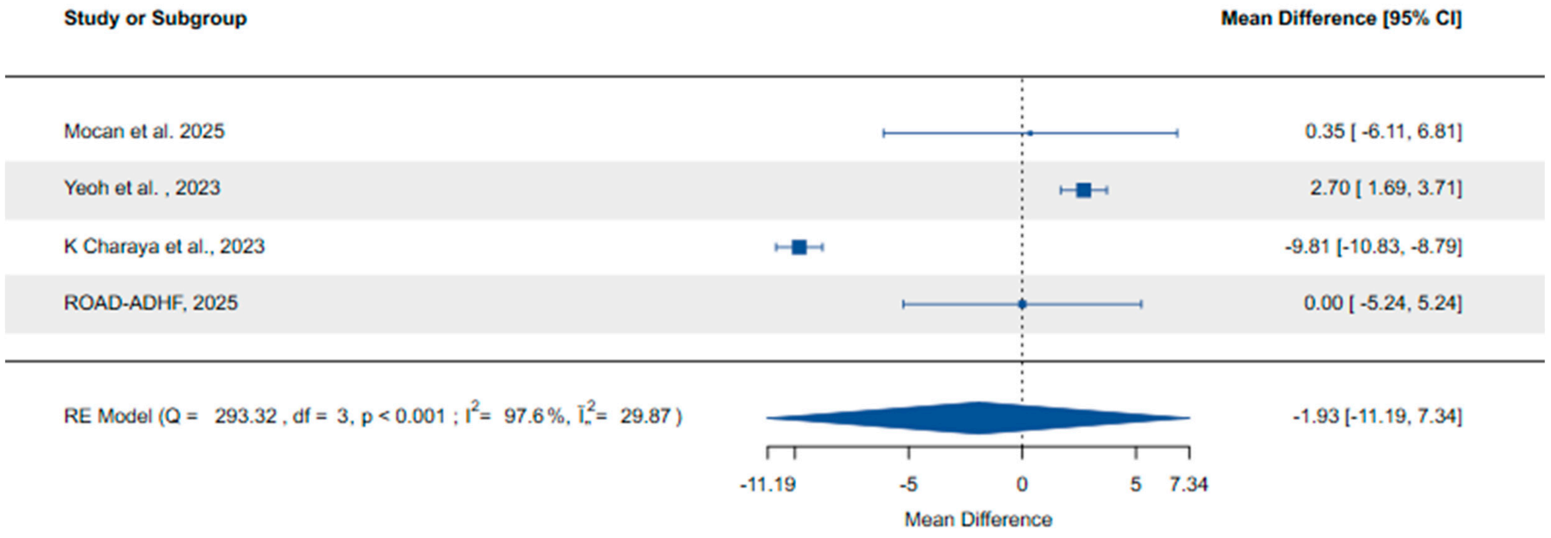

Supplementary Figure S14. Forest plot for eGFR change.

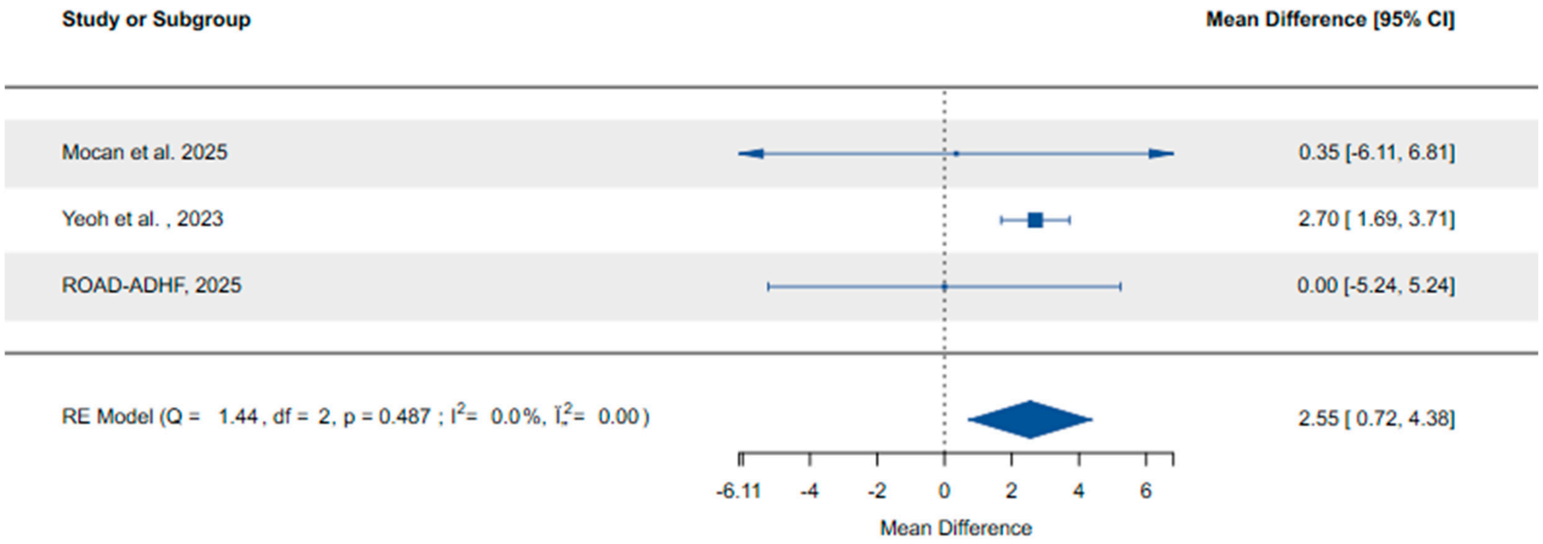

Supplementary Figure S15. Leave-one-out sensitivity analysis for eGFR change.

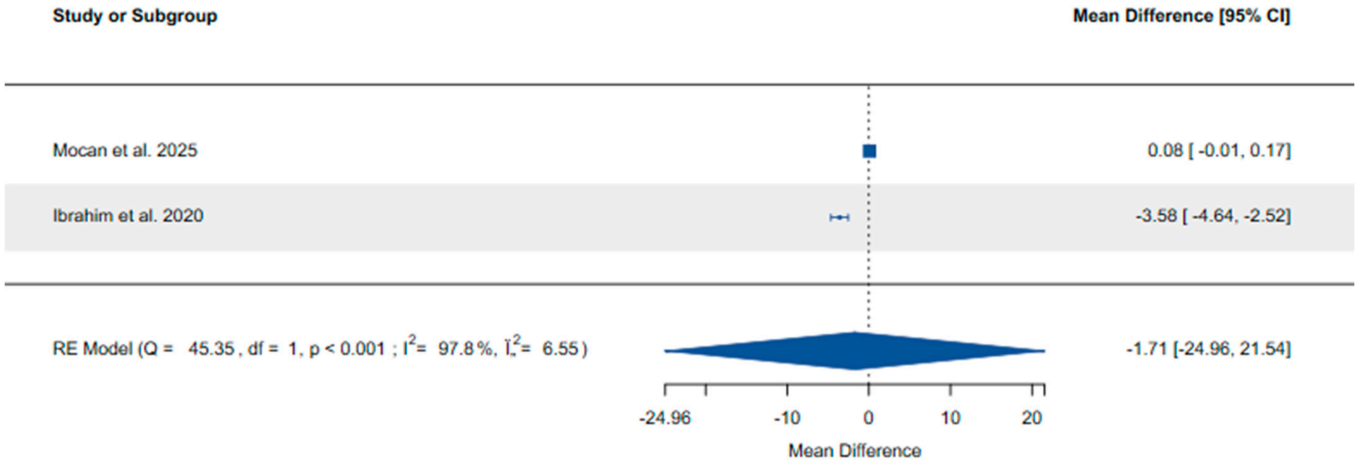

Supplementary Figure S16. Forest plot for serum creatinine change.

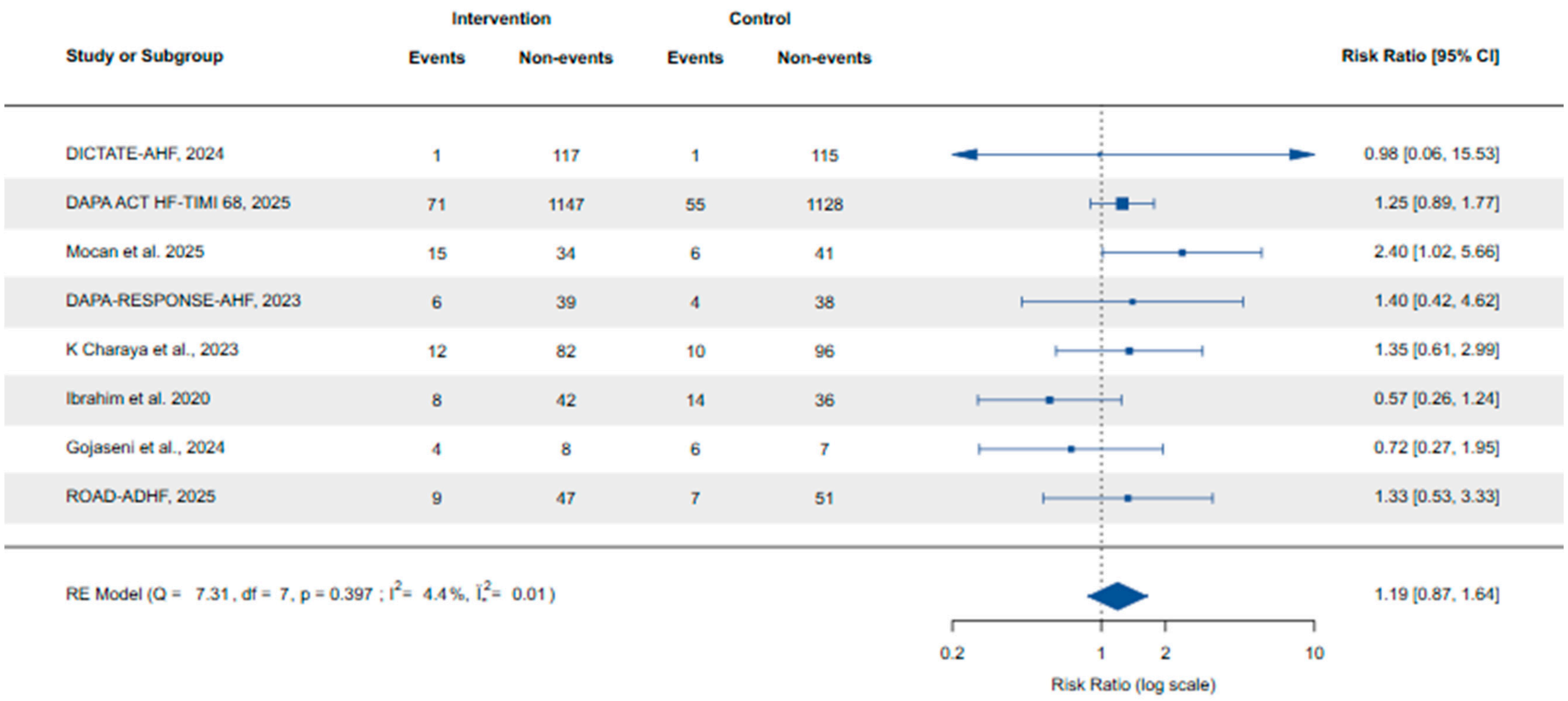

Supplementary Figure S17. Forest plot for acute kidney injury.

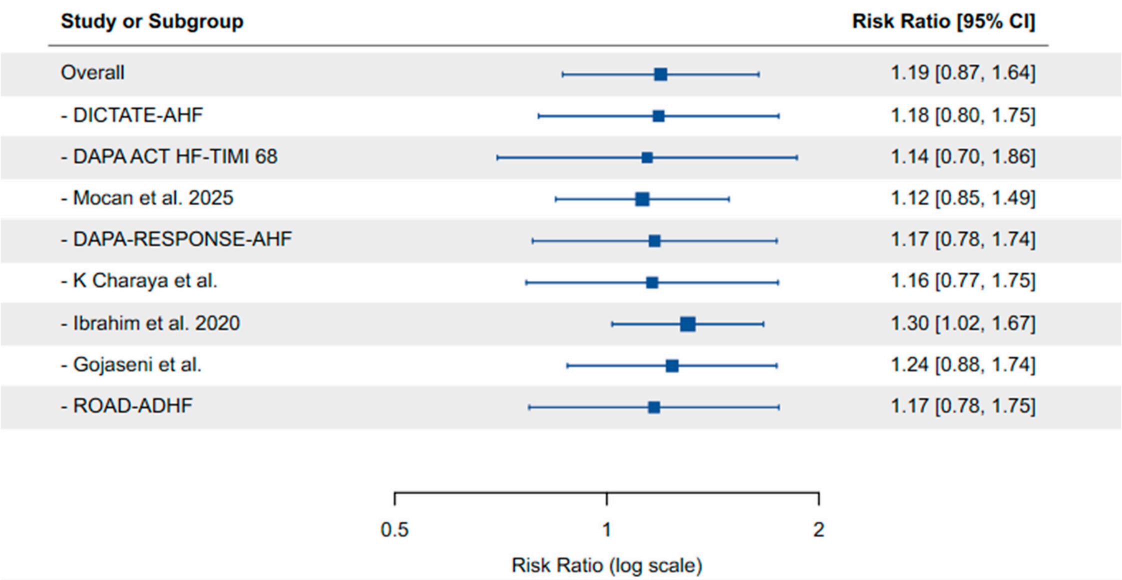

Supplementary Figure S18. Leave-one-out sensitivity analysis for acute kidney injury.

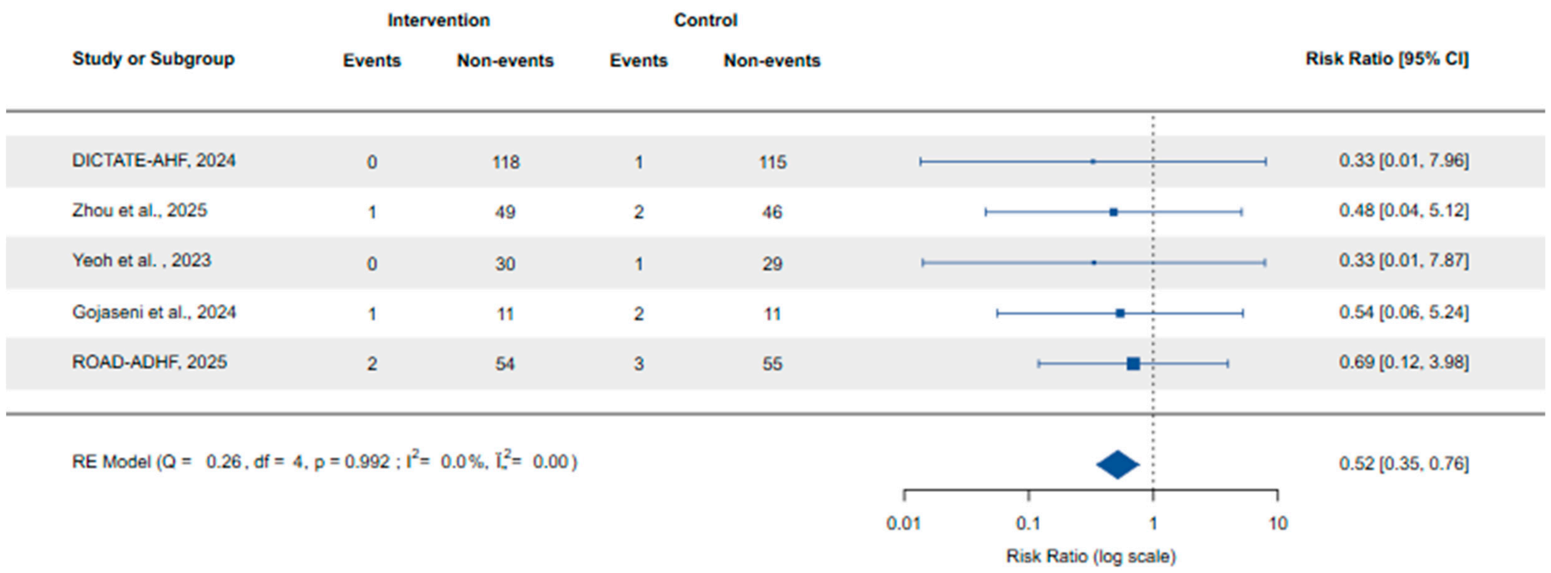

Supplementary Figure S19. Forest plot for urinary tract infection. (p=0.009)

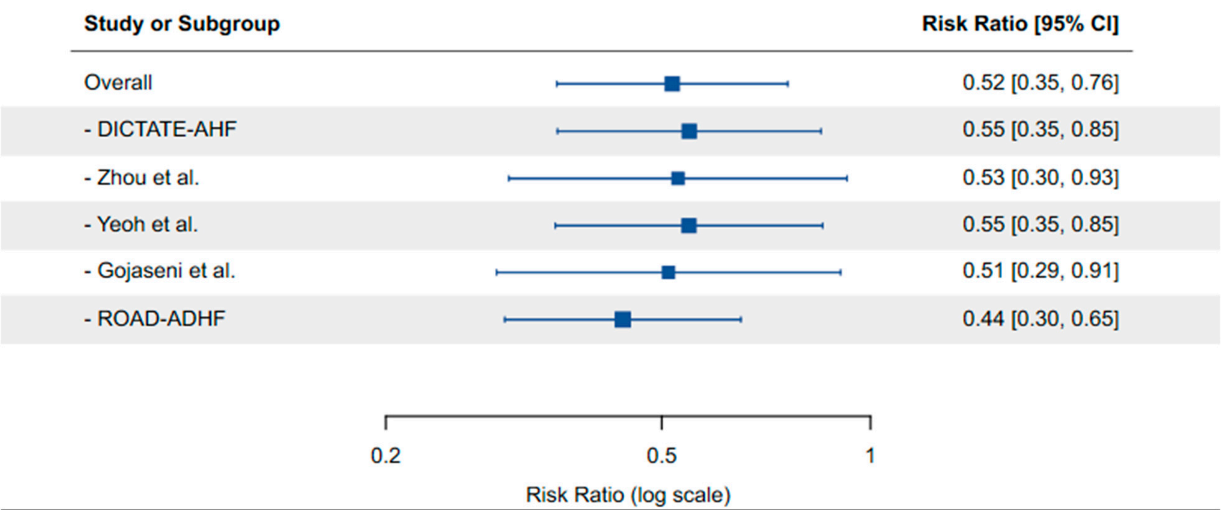

Supplementary Figure S20. Leave-one-out sensitivity analysis for urinary tract infection.

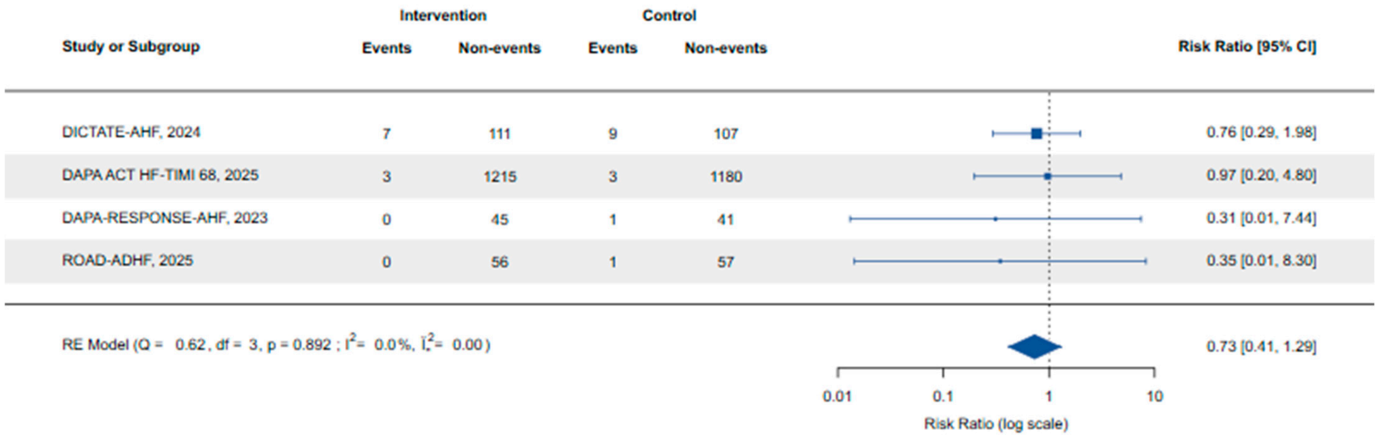

Supplementary Figure S21. Forest plot for hypoglycemia.

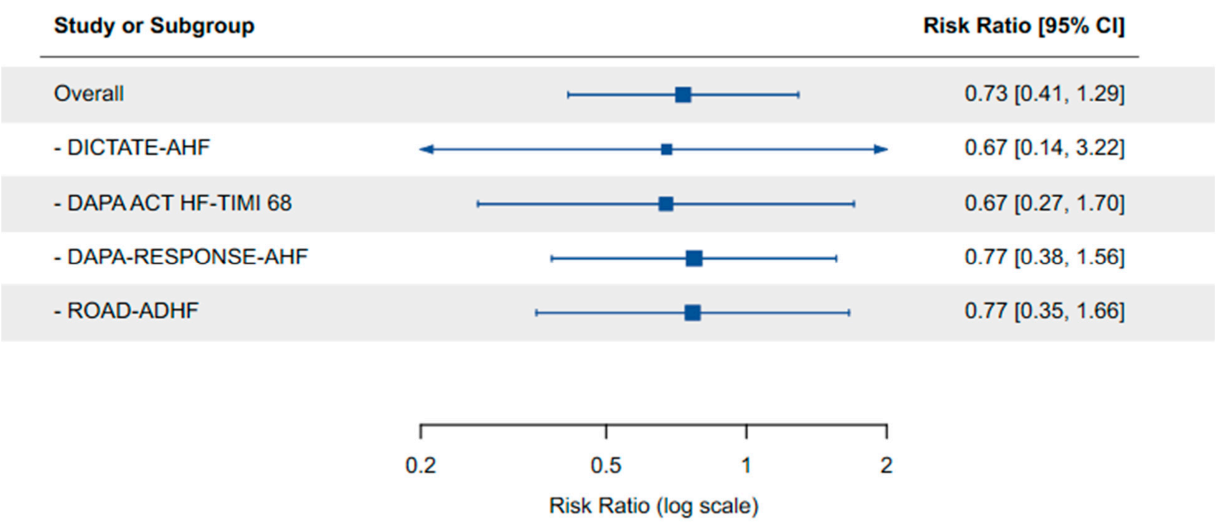

Supplementary Figure S22. Leave-one-out sensitivity analysis for hypoglycemia.

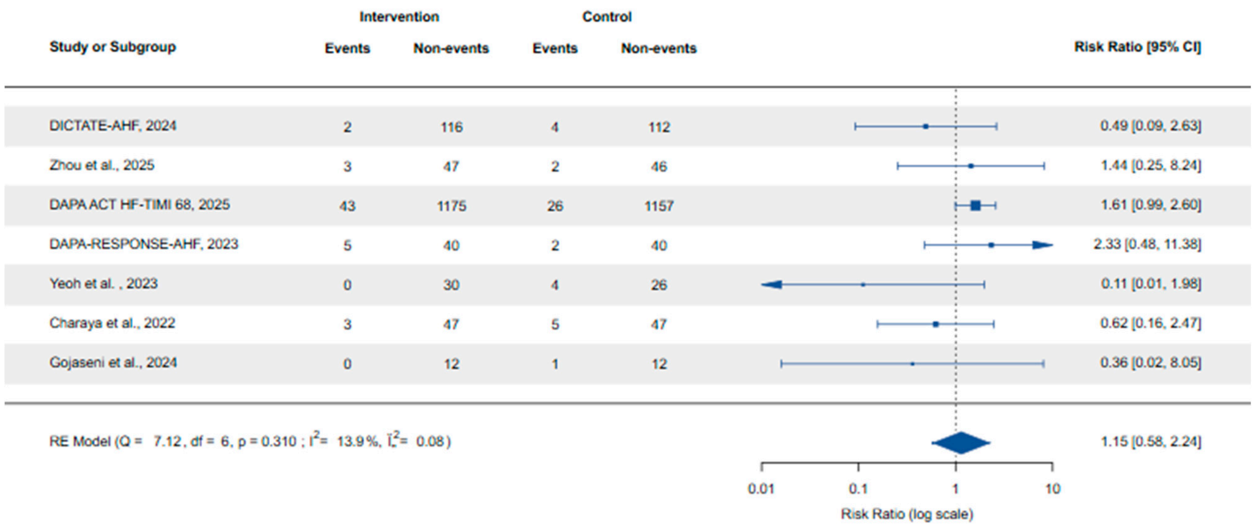

Supplementary Figure S23. Forest plot for hypotension.

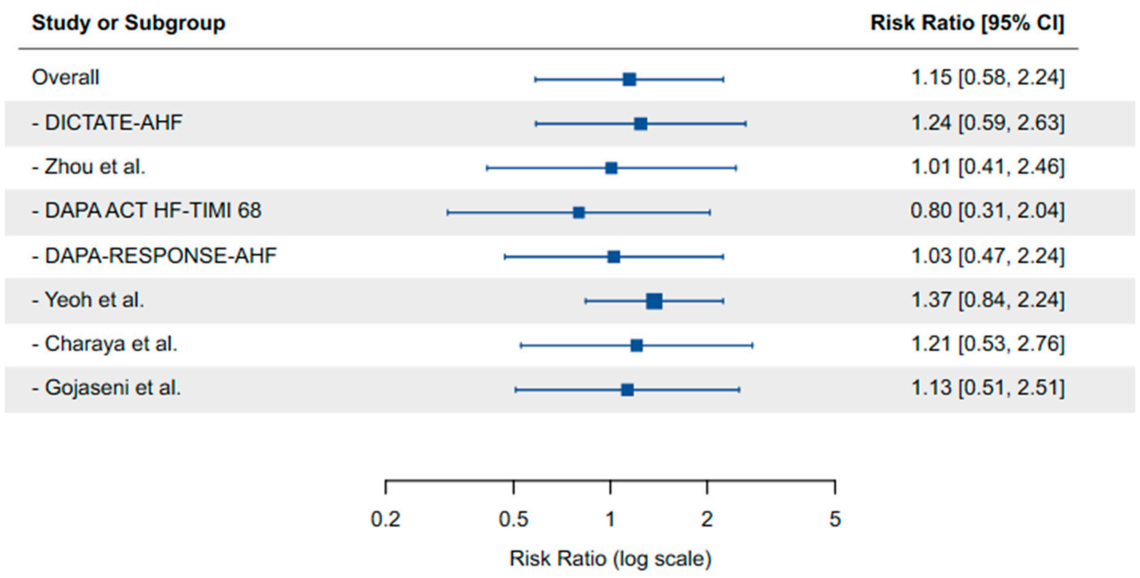

Supplementary Figure S24. Leave-one-out sensitivity analysis for hypotension.

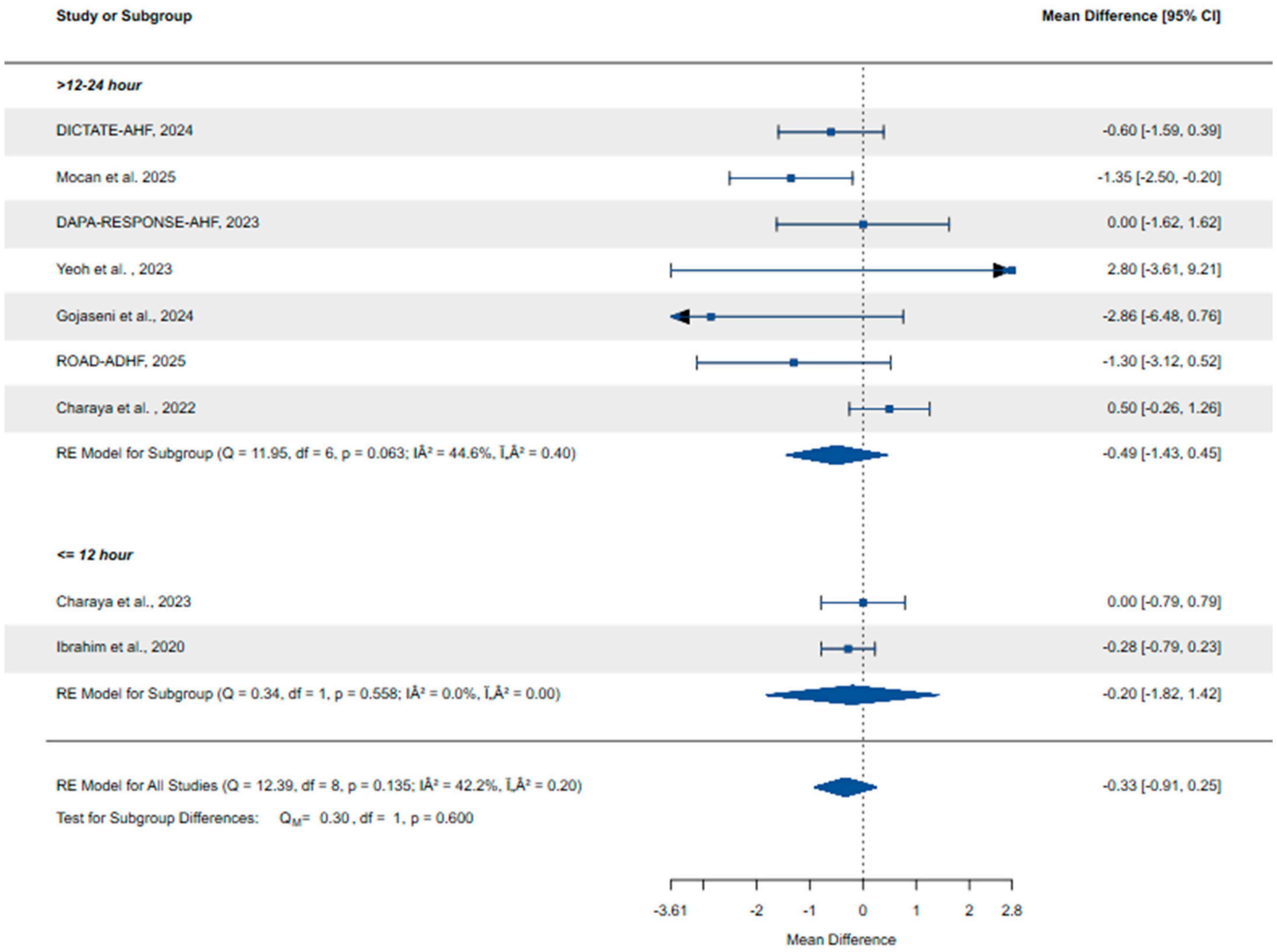

Supplementary Figure S25. Subgroup analysis of length of hospital stay according to the 12-hour dapagliflozin initiation threshold.

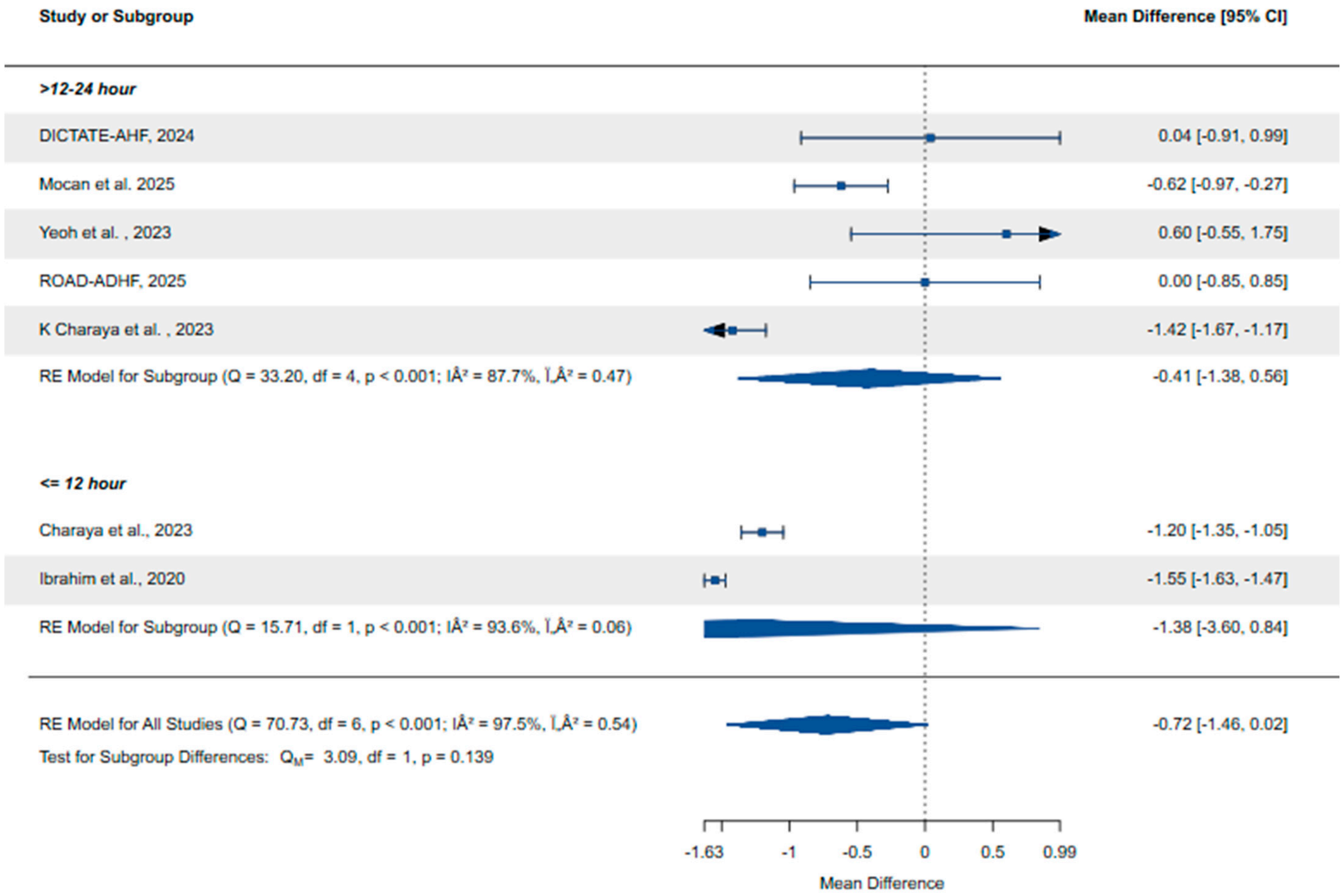

Supplementary Figure S26. Subgroup analysis of weight/BMI change according to the 12-hour dapagliflozin initiation threshold.
